# Instability of CTG Repeats is Governed by the Position of a DNA Base Lesion through Base Excision Repair

**DOI:** 10.1371/journal.pone.0056960

**Published:** 2013-02-26

**Authors:** Yanhao Lai, Meng Xu, Zunzhen Zhang, Yuan Liu

**Affiliations:** 1 Department of Chemistry and Biochemistry, Florida International University, Miami, Florida, United States of America; 2 Department of Environmental and Occupational Health, West China School of Public Health, Sichuan University, Chengdu, People’s Republic of China; Institute of Molecular Genetics IMG-CNR, Italy

## Abstract

Trinucleotide repeat (TNR) expansions and deletions are associated with human neurodegeneration and cancer. However, their underlying mechanisms remain to be elucidated. Recent studies have demonstrated that CAG repeat expansions can be initiated by oxidative DNA base damage and fulfilled by base excision repair (BER), suggesting active roles for oxidative DNA damage and BER in TNR instability. Here, we provide the first evidence that oxidative DNA damage can induce CTG repeat deletions along with limited expansions in human cells. Biochemical characterization of BER in the context of (CTG)_20_ repeats further revealed that repeat instability correlated with the position of a base lesion in the repeat tract. A lesion located at the 5′-end of CTG repeats resulted in expansion, whereas a lesion located either in the middle or the 3′-end of the repeats led to deletions only. The positioning effects appeared to be determined by the formation of hairpins at various locations on the template and the damaged strands that were bypassed by DNA polymerase β and processed by flap endonuclease 1 with different efficiency. Our study indicates that the position of a DNA base lesion governs whether TNR is expanded or deleted through BER.

## Introduction

Trinucleotide repeat (TNR) expansions are identified as the cause of more than 40 neurodegenerative diseases [1], and their deletions are implicated in cancer development [2]. TNRs associated with human diseases include (CAG)_n_/(CTG)_n_, (CTG)_n_/(CAG)_n_, (CGG)_n_/(CCG)_n_, and (GAA)_n_/(TTC)_n_. Expansions of these repeats are responsible for Huntington’s disease (HD), spinocerebellar ataxia, myotonic dystrophy type 1 (DM1), fragile X syndrome, and Friedreich’s ataxia [1]. Epidemiological studies also suggest a correlation between CAG repeat deletions in the androgen receptor and prostate and ovarian cancers [2], [3], implying that TNR deletions are equally as important as TNR expansions in causing human diseases.

Over the past 20 years, substantial progress has been made in understanding the mechanisms underlying TNR expansions and deletions using model systems such as bacteria [4], [5], yeast [6], mammalian cells [7], and mouse models of TNR-related human diseases [8]. TNR instability is considered to be mediated by the formation of a series of non-B form DNA secondary structures and their metabolism by DNA replication [9], repair [10], and recombination [11]. Typical non-B form DNA structures include hairpins and tetraplexes that are usually generated by CAG, CTG, and CGG repeats due to their propensity of self-base pairing [12]. Hairpin structures generated on a strand with newly synthesized DNA usually cause expansions, whereas hairpins formed on a template strand usually cause repeat deletions [13]. Therefore, factors that can facilitate the formation and stability of TNR hairpins could lead to TNR instability. For example, the length of a TNR tract appears to be critical for TNR expansion. It has been found that expansions can occur when the repeat length is greater than 35−42 units. This is called the threshold of TNR expansions [14] that presumably allows the formation of stable secondary structures, and further evades cellular repair mechanisms for removing the structures [15]. However, the outcomes for TNR expansions or deletions are ultimately determined by DNA replication [1], [14], [16], [17], repair, and double-stranded DNA repair-mediated recombination [18], during which TNR secondary structures are processed for their genome integration [19], [20]. Thus, the stability of TNRs may be modulated by the interactions between dynamic DNA structures and replication, repair, and recombination machinery.

One of the most important features of TNRs is that they all are composed of a stretch of guanines, which allow them to become the hotspots of oxidative DNA damage. A link between oxidative DNA damage and TNR instability has been established in bacteria [5], [21], mammalian cells, tissues [22], [23], and mouse models [24]. Exposure of bacteria to hydrogen peroxide (H_2_O_2_) increased the deletions of TNRs [5]. H_2_O_2_ significantly increased large deletions of CAG/CTG tracts in mouse kidney cells [23], whereas it induced small CAG repeat expansions in human lymphocytes [22]. Consistent with these observations, an increased level of 8-oxoguanine (8-oxoG) was associated with age-dependent CAG repeat expansions in the striatum of HD transgenic mouse models [22], [25]. In addition, potassium bromate, an environmental oxidative DNA damaging agent, increased the level of 8-oxoG and CGG repeat expansions in the germ cells of fragile X syndrome pre-mutation mice [24]. Thus, oxidative DNA damage is actively involved in causing TNR instability, and its repair appears to play crucial roles in modulating TNR instability. This hypothesis is supported by a recent finding that 8-oxoG DNA glycosylase (OGG1), an enzyme that specifically removes 8-oxoG, is required for the age-dependent somatic CAG repeat expansions in the striatal neurons of a HD mouse model [22]. Moreover, an essential enzyme of base excision repair (BER), DNA polymerase β (pol β) binds to CAG repeats *in vivo* in the striatum of HD mice [26], suggesting an important role of pol β-mediated BER in modulating CAG repeat instability. Our previous study demonstrated that removal of an 8-oxoG in the context of CAG repeats by OGG1 induced single-stranded DNA (ssDNA) breaks leading to DNA strand slippage and the formation of a 5′-hairpin [27]. This disrupts efficient long-patch BER that is mediated by the "hit-and-run" mechanism through pol β and flap endonuclease 1 (FEN1) [27], [28], thereby resulting in an inefficient long-patch BER that involves pol β multi-nucleotide gap-filling synthesis and FEN1 alternate flap cleavage [27]. In support of this possibility, low levels of FEN1 along with normal levels of pol β in the striatum of HD mice were associated with CAG repeat expansions [26]. Thus, it appears that inefficient BER is associated with TNR expansions.

Oxidative DNA damage may preferentially occur at specific locations of TNR tracts. This could modulate DNA repair efficiency and affect the outcomes of TNR instability. Oxidized DNA base lesions are preferentially induced at the loop region of a hairpin by a DNA damaging agent directly [29] and by repositioning of the lesions located in the stem of a repeat hairpin [30]. However, the lesion at this specific location was found to be resistant to OGG1 activity [31], allowing its escape from BER, leading to multiple rounds of "toxic oxidation cycles" for TNR expansion [1], [31]. An abasic lesion located at the 5′-end of CAG repeats was removed by BER with a much lower efficiency than the abasic lesion located either at the 3′-end or in the middle of the repeats [32]. These results suggest that the positions of an oxidized base lesion in TNR tracts alter its repair efficiency that modulates accumulation of ssDNA breaks and hairpin structures at specific locations. Consequently, this would direct the damage repair path towards repeat expansions or deletions.

Here, we asked several important questions with regard to the positions of DNA base lesions and TNR instability. Can a specific location of a base lesion determine whether TNR repeat tracts are expanded or deleted through BER? If so, how are BER enzymes involved in mediating the positioning effect of base lesions, and how can TNR instability be regulated when multiple base lesions occur in TNR tracts simultaneously? In this study, we show for the first time that oxidative DNA damaging agents induce various sizes of CTG repeat deletions and limited sizes of expansions in human cells. We demonstrate that the position of an oxidative base lesion governs the instability of CTG repeats through the imbalanced activities of pol β DNA synthesis and FEN1 alternate flap cleavage. Our study provides new insights into the molecular mechanisms underlying TNR expansion and deletion induced by oxidative DNA damage.

## Materials and Methods

### Materials

Potassium chromate (K_2_CrO_4_, purity ≥98.0%) and potassium bromate (KBrO_3_, purity ≥98.0%) were obtained from Alfa Aesar (Ward Hill, MA). Thirty percent (w/w) H_2_O_2_ was from BDH (London, England). Dulbecco’s modified eagle medium (DMEM), fetal bovine serum (FBS), L(+)-glutamine, and 0.25% trypsin-EDTA were purchased from Life Technologies (Grand Island, NY). DNA oligonucleotides were synthesized by Integrated DNA Technologies Inc. (Coralville, IA). The radionucleotide [γ−^32^P] ATP (6000 mCi/mmol) and cordycepin 5′-triphosphate 3′- [α−^32^P] (5000 mCi/mmol) were purchased from PerkinElmer Inc. (Boston, MA). Micro Bio-Spin 6 chromatography columns were from Bio-Rad (Hercules, CA). Deoxynucleoside 5′-triphosphates (dNTPs) were from Roche Diagnostics (Indianapolis, IN). T4 polynucleotide kinase and terminal nucleotidyltransferase were from Fermentas (Glen Burnie, MD). Mung Bean Nuclease was from Epicenter (Madison, WI). All other reagents were purchased from Sigma-Aldrich (St. Louis, MO) and Fisher Scientific (Pittsburgh, PA). Purified recombinant human apurinic/apyrimidinic endonuclease 1 (APE1), pol β, FEN1, and DNA ligase I (LIG I) were generous gifts from Dr. Samuel Wilson at the National Institute of Environmental Health Sciences, National Institutes of Health (Research Triangle Park, NC) or were expressed and purified as described previously [28].

### Oligonucleotide Substrates

DNA oligonucleotide substrates containing a tetrahydrofuran (THF), an abasic site analog in the context of (CTG)_20_ repeats were used to mimic an oxidized abasic site. The guanines in the first, tenth, twentieth, or both the first and eleventh CTG unit were substituted with a THF residue. Substrates were constructed by annealing an oligonucleotide strand with one or two base lesions to its template strand at a molar ratio of 1∶2. A DNA fragment that contained (CTG)_20_ without any DNA base lesions was used as a size marker for DNA fragment analysis. The sequences and descriptions of the oligonucleotides are shown in Supplemental Table S1.

### Cell Culture and Transfection of (CTG)_35_/(CAG)_35_ and (CTG)_20_/(CAG)_20_-containing Plasmids

Human embryonic kidney (HEK) 293-H cells (Life Technologies, Grand Island, NY) were cultured in DMEM supplemented with 10% FBS and 4 mM L(+)-glutamine at 37°C under 5% CO_2_. A plasmid containing (CTG)_35_/(CAG)_35_ or (CTG)_20_/(CAG)_20_ repeats was constructed by inserting a fragment containing a (CTG)_35_/(CAG)_35_ or (CTG)_20_/(CAG)_20_ tract flanked by the 5′- and the 3′-side random DNA sequences into pcDNA3.1/CT-GFP-TOPO vector (Life Technologies), respectively. A DNA fragment containing a random sequence with the same length as the (CTG)_35_/(CAG)_35_ repeat-containing fragment (225 nt) or (CTG)_20_/(CAG)_20_ repeat-containing fragment (100 nt) was cloned into pcDNA3.1/CT-GFP-TOPO for constructing the plasmids used as the random sequence control. Plasmids (12 µg) were pre-incubated with 36 µl lipofectamine 2000 (Life Technologies), for 20 min at room temperature. The mixture of plasmids and lipofectamine was subsequently transferred to the medium supplied for culturing 4×10^5^ HEK293-H cells. Cellular transfection efficiency was determined using a fluorescent microscope (Leica, Wetzlar, Germany). For all the experiments, the transfection efficiency was greater than 95%.

### Measurement of Instability of (CTG)_35_/(CAG)_35_ and (CTG)_20_/(CAG)_20_ Induced by Oxidative DNA Damage in HEK293-H cells

Instability of (CTG)_35_/(CAG)_35_ or (CTG)_20_/(CAG)_20_ repeats induced by oxidative DNA damage was examined by treating 4×10^5^ HEK293-H cells, transfected with the (CTG)_35_/(CAG)_35_ or (CTG)_20_/(CAG)_20_-containing plasmids, using three well known environmental and endogenous oxidative DNA-damaging agents, KBrO_3_, K_2_CrO_4_, and H_2_O_2_ at concentrations of 30 mM, 300 µM, and 1 mM, respectively for 2 hr. Cells were washed twice with phosphate-buffered saline (PBS), supplied with fresh medium, and grown for 2 days to allow recovery from DNA damage. The treatment was repeated three times before cells were harvested. In the control experiment, HEK293-H cells transfected with plasmids that contain random DNA sequences were treated by three DNA damaging agents under the same conditions used for treatment of cells bearing repeat-containing plasmids. At the end of the experiments, cells were trypsinized using 0.25% trypsin-EDTA and harvested by centrifugation at 3000 rpm for 15 min. Plasmids were isolated from cells using Qiagen Miniprep Kits (Qiagen, Valencia, CA), dissolved in Tris-EDTA (TE) buffer (10 mM Tris-HCl, pH 7.5, and 1 mM EDTA), and stored at −20°C for subsequent size analysis. Untreated cells served as a negative control. The experiments were repeated at least 3 times.

### *In Vitro* BER in Mouse Embryonic Fibroblast Cell Extracts

Pol β null (pol β^−/−^) and wild type (pol β^+/+^) mouse embryonic fibroblasts (MEFs) were grown to near confluence. Cells were washed twice with PBS, harvested by cell scrapers, and centrifuged at 3000 rpm for 15 min. Cell extracts were made as described previously [33] and were dialyzed into BER reaction buffer containing 50 mM Tris-HCl, pH 7.5, 50 mM KCl, 0.1 mM EDTA, 0.1 mg/ml bovine serum albumin, and 0.01% Nonidet P-40. Substrates were pre-incubated with 50 nM purified APE1 at 37°C for 30 min, and completely converted into ssDNA break intermediates for subsequent BER reactions. *In vitro* BER of a THF in pol β^−/−^ and pol β^+/+^ cell extracts was performed by incubating APE1 precut (CTG)_20_ repeat-containing substrate with 60 µg cell extracts under the conditions described previously [27]. Reaction mixtures were assembled on ice and incubated at 37°C for 30 min. BER reactions were terminated by transferring to 95°C for 5 min. Reaction mixtures were subsequently digested with protease K at 55°C for 30 min. Repair intermediates and products were precipitated and dissolved in stopping buffer containing 95% formamide and 2 mM EDTA, and were separated by 15% urea-denaturing polyacrylamide gel electrophoresis (PAGE). Repair products were further isolated from the gel and eluted with TE buffer through rotation at room temperature overnight. The products were precipitated with ethanol, dissolved in TE buffer, and stored at −20°C for subsequent size analysis.

### *In vitro* BER Reconstituted with Purified Enzymes

BER of ssDNA break intermediates was reconstituted by incubating 50 nM purified APE1, 10 nM pol β, 10 nM FEN1, and 5 nM LIG I with 25 nM (CTG)_20_ repeat-containing substrates with one or two THF residues. The 20 µl reaction mixture contained BER buffer with 50 µM dNTPs, 5 mM Mg^2+^, 2 mM ATP, and indicated concentrations of BER enzymes and substrates. Reaction mixtures were assembled on ice, and incubated at 37°C for 15 min. Reactions were terminated by transferring to 95°C for 5 min in stopping buffer. Repair products and intermediates were separated by 15% urea-denaturing PAGE. Repair products were isolated from the gel and eluted with TE buffer through rotation at room temperature overnight. The products were precipitated with ethanol, dissolved in TE buffer, and stored at −20°C for subsequent sizing analysis.

### Probing of Hairpin Structures by Mung Bean Nuclease Digestion

Hairpin formation on the damaged and template strands of (CTG)_20_-containing substrates were probed by Mung Bean Nuclease digestion. Substrates (200 nM) containing one or two THF residues at different locations of (CTG)_20_ repeats were pre-cut by 10 nM APE1 and were subjected to digestion with 1 U Mung Bean Nuclease at 37°C for 1, 2, 3, 5, and 8 min. The 10-µl reaction was conducted in buffer containing 30 mM sodium acetate (pH 4.6), 50 mM NaCl, 1 mM zinc acetate, and 0.01% Triton X-100. Enzymatic reactions were terminated by 2 µg proteinase K digestion at 55°C for 30 min. Reaction mixtures were subjected to 95°C for 10 min to denature DNA. Substrates and digestion products were separated by 15% urea-denaturing PAGE and detected by a Pharos FX Plus PhosphorImager from Bio-Rad. Synthesized DNA size markers were used to indicate the size of nuclease cleavage products.

### Enzymatic Activity Assay

Pol β DNA synthesis during BER of ssDNA break intermediates was measured by using 25 nM oligonucleotide substrates containing (CTG)_20_ with one or two THF residues as illustrated in Supplemental Table S1. Pol β activity was examined at 37°C in a 20 µl reaction mixture that contained BER reaction buffer with 50 µM dNTPs and 5 mM Mg^2+^. FEN1 cleavage activity in the absence or presence of pol β was examined under the same conditions used for determining pol β activity. Repair intermediates and products were separated by 15% urea-denaturing PAGE and detected by a PhosphorImager. Synthesized size markers for illustrating the size of pol β DNA synthesis products or FEN1 cleavage products were run in parallel with repair products.

### Sizing Analysis of CTG Repeats by DNA Fragment Analysis and PeakScanner Software

The size of repaired products was determined by capillary electrophoresis using an ABI 3130XL Genetic Analyzer (Applied Biosystems, Foster City, CA) and DNA fragment analysis with PeakScanner version 1.0 software (Applied Biosystems, Foster City, CA) with assistance from the DNA Sequencing Core of Florida International University. A 225 nt- or 100 nt-DNA fragment in plasmids containing (CTG)_35_/(CAG)_35_ repeats or (CTG)_20_/(CAG)_20_ repeats or random sequences was amplified by PCR using a forward primer with a 5′–6-carboxyfluorescein tag and a reverse primer (Supplementary Table S1). PCR amplification conditions were as follows: 95°C for 10 min, 1 cycle; 95°C for 30 s, 50°C (for repeats) or 55°C (for random sequences) for 30 s, and 72°C for 1.5 min, 35 cycles; final extension at 72°C for 1 hr. Size standards, MapMarker 1000 (Bioventures, Murfreesboro, TN) were run in parallel with PCR-amplified repair products.

## Results

### CTG Repeat Deletions and Expansions Induced by Oxidative DNA Damage in Human Cells

To determine how oxidative DNA damage may affect TNR instability in human somatic cells, we initially examined the effects of environmental and endogenous oxidative DNA damaging agents on CTG repeat instability in HEK293-H cells. We reasoned that these agents can result in a stretch of 8-oxoGs and ssDNA breaks in TNRs that lead to accumulation of ssDNA break intermediates, DNA slippage, and the formation of hairpin structures on both the damaged and template strands of TNR tracts. The repair of ssDNA break intermediates including hairpin structures could allow integration of the hairpins into the genome, thereby causing expansions and deletions.

**Figure 1:**
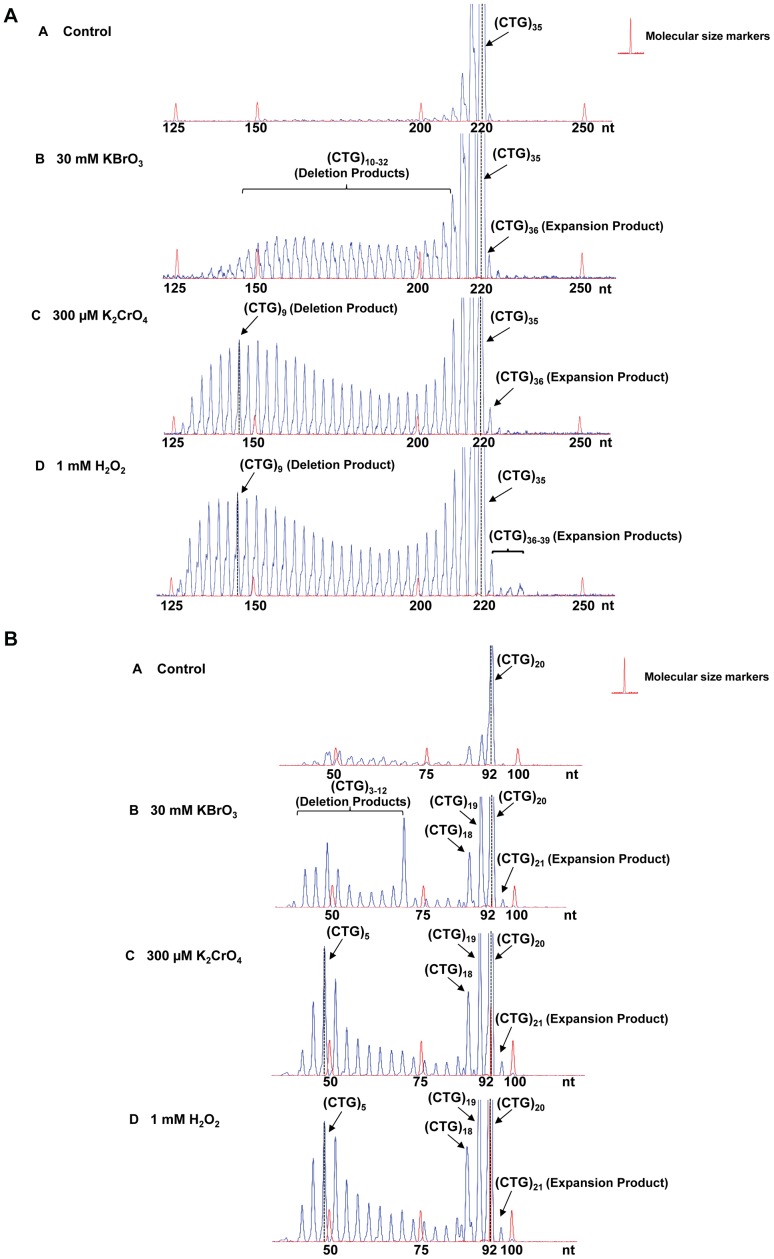
CTG repeat instability induced by oxidative DNA damage in human cells. (**A**) Plasmids containing (CTG)_35_ repeats (12 µg) were transfected into HEK293-H cells as described in the Materials and Methods. Panel **A** represents the result from untreated cells. Panels **B**, **C,** and **D** represent the results from the cells treated with KBrO_3_, K_2_CrO_4,_ and H_2_O_2_, respectively. (**B**) HEK293-H cells were transfected with plasmids containing (CTG)_20_ repeats (12 µg). Panel **A** illustrates the result from untreated cells. Panels **B**, **C,** and **D** represent the results from the cells treated with KBrO_3_, K_2_CrO_4,_ and H_2_O_2_, respectively. The repaired products are illustrated as peaks. The height of a peak indicates the abundance of a specific repair product. The sizes of repair products are illustrated in nucleotides. Size standards are indicated.

To explore this possibility, we examined the instability of (CTG)_35_/(CAG)_35_ and (CTG)_20_/(CAG)_20_ repeats induced by oxidative DNA damaging agents, KBrO_3,_ K_2_CrO_4_,and H_2_O_2_ [34], [35] in HEK293-H cells. The results showed that the length of (CTG)_35_/(CAG)_35_ repeats in the untreated cells varied between 33 to 35 repeat units, although a small portion of plasmids containing 32 and 36 repeats were detected (Figure 1A, panel A). The length of (CTG)_20_/(CAG)_20_ repeats in the untreated cells ranged from (CTG)_18_ to (CTG)_20_ repeats. Exposure of cells to 30 mM KBrO_3_ resulted in a series of deletion products with (CTG)_10_ to (CTG)_32_ repeats for (CTG)_35_-containing plasmids (Figure 1A, panel B) and deletion products with (CTG)_3_ to (CTG)_6_ or (CTG)_12_ or (CTG)_18_ to (CTG)_19_ repeats for (CTG)_20_-containing plasmids (Figure 1B, panel B). Thus, bromate-induced DNA damage led to deletion of (CTG)_35_ repeats by 3 to 25 repeat units and deletion of (CTG)_20_ by 1–17 repeat units. Three hundred micromoles of K_2_CrO_4_ and 1 mM H_2_O_2_ led to deletion products with repeat length mainly ranging from (CTG)_4_ to (CTG)_32_ for (CTG)_35_ repeats (Figure 1A, panels C and D) and (CTG)_4_ to (CTG)_19_ for (CTG)_20_ repeats (Figure 1B, panels C and D). In addition, all of the DNA damaging agents led to small amounts of expansion products with (CTG)_36_ to (CTG)_39_ repeats for (CTG)_35_ repeat-containing plasmids (Figure 1A, panels B, C, and D) and an expansion product with (CTG)_21_ repeats for (CTG)_20_ repeat-containing plasmids (Figure 1B, panels B, C, and D).

Bromate, chromate and hydrogen peroxide failed to induce any length change in a 225 nt- and 100 nt-fragment that contained random DNA sequence (Supplementary Figure S1) indicating that oxidative DNA damage-induced CTG repeat instability was TNR sequence specific.

Interestingly, KBrO_3_-induced deletion products from (CTG)_35_ repeats exhibited a pattern with even size distribution, which suggests that a single 8-oxoG may be induced at different repeats in a randomized manner. KBrO_3_-induced deletion products from (CTG)_20_ repeats contained (CTG)_18–19_, (CTG)_12_ and (CTG)_3–6_ repeats that correspond to small, middle and large size deletion, respectively, suggesting that the agent resulted in a similar deletion pattern in (CTG)_20_ repeats as the one in (CTG)_35_ repeats. In contrast, K_2_CrO_4_ and H_2_O_2_ predominantly induced the deletions with a peak size of (CTG)_9_ or (CTG)_10_ repeats. The size distribution pattern of deletions and expansions suggests that a specific oxidative DNA damaging agent may induce a single or multiple base lesions/ssDNA breaks at specific positions in a CTG repeat tract, preferentially leading to either repeat deletions or expansions.

We designated the damage position-specific effect on the instability of CTG repeats as the “DNA damage positioning effect.” Because oxidative DNA damage is mainly repaired by BER, it is possible that the positioning effects of oxidative DNA damages are accomplished through BER of oxidative DNA base lesions in the context of CTG repeats. To test this possibility, we examined the effects of the position and the number of DNA base lesions on CTG repeat instability during *in vitro* cell extract-based and reconstituted BER.

### A Specific Location of a DNA Base Lesion on (CTG)_20_ Repeats Correlated with Repeat Expansion or Deletion

The position of a DNA base lesion or ssDNA break in CTG repeats may be classified as at the 5′-end, in the middle, or at the 3′-end of the repeat tract. To examine how a base lesion at these positions may modulate repeat instability, we used a series of (CTG)_20_ repeat-containing substrates with an abasic lesion represented by a THF residue that substituted the guanine of the first, tenth, and twentieth CTG unit. These substrates mimic the scenarios wherein a single oxidized base lesion occurs at the 5′-end, in the middle, and at the 3′-end of a (CTG)_20_ repeat tract, respectively. A substrate containing two THF residues embedded in the first (the 5′-end) and the eleventh CTG (the middle) of (CTG)_20_ repeats was used to mimic a situation in which more than one DNA base lesion occurs simultaneously in the repeat tract.

**Figure 2:**
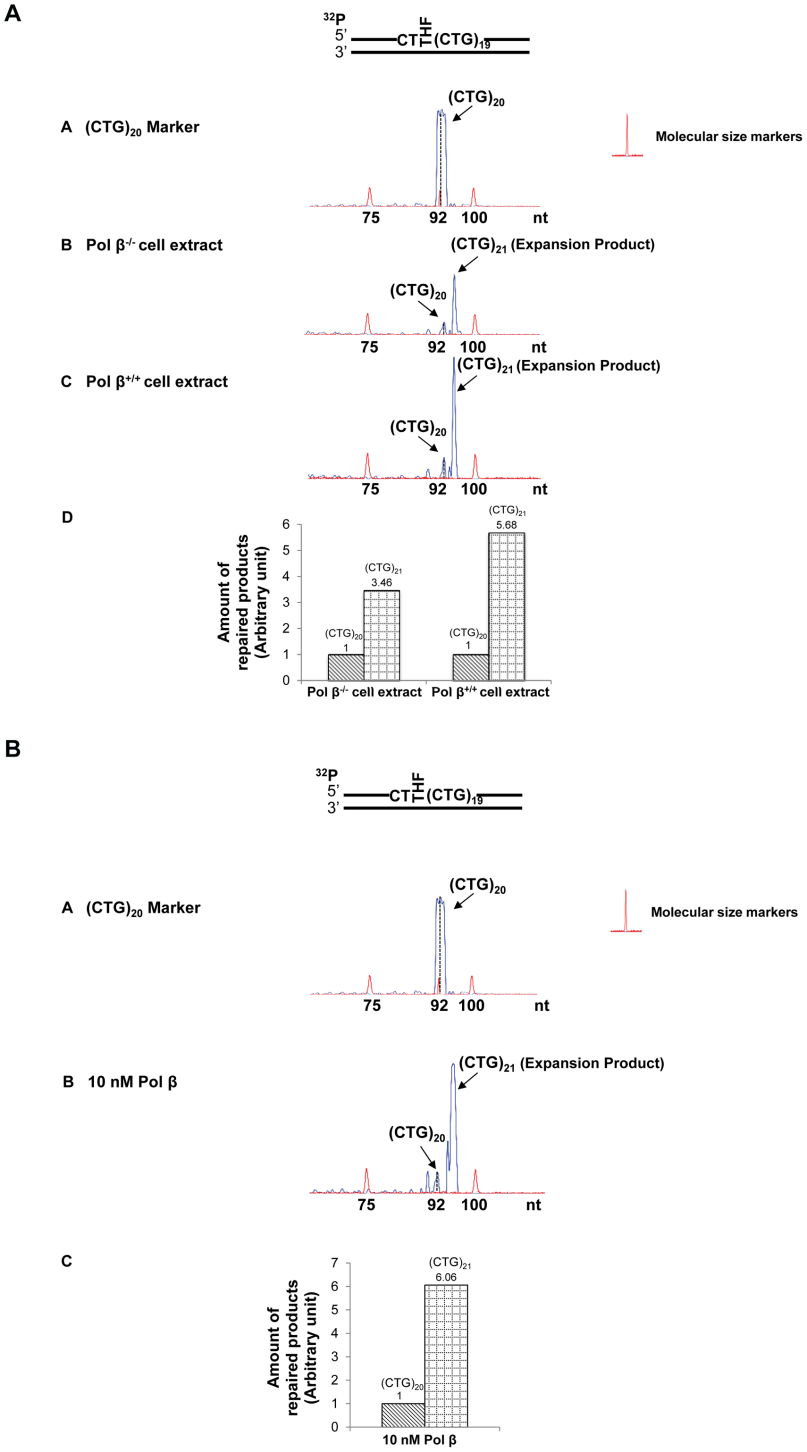
CTG repeat expansion resulting from an abasic lesion located at the 5′-end of (CTG)_20_ repeats. (**A**) A substrate containing (CTG)_20_ repeats with a THF inserted for substituting the guanine of the first CTG was incubated with cell extracts of pol β^−/−^ or pol β^+/+^ MEFs under the conditions described in the Materials and Methods. Panel **A** represents the result of PCR amplification of a DNA marker containing (CTG)_20_ repeats without any damage. Panels **B** and **C** illustrate the results from BER mediated by pol β^−/−^ and pol β^+/+^ MEFs extracts. Panel **D** represents the quantitative analysis of the results of panels **B** and **C**. (**B**) The THF at the 5′-end of (CTG)_20_ repeats was repaired by BER reconstituted with 10 nM purified pol β as described in the Materials and Methods (panel **B**). Panel **A** illustrates the result of PCR amplification of a (CTG)_20_ repeat-containing marker without any DNA damage, and panel **C** illustrates the quantitative analysis of the results from panel **B**. Substrates were ^32^P-labeled at the 5′-end of the damaged strand as indicated. Size standards are indicated.

**Figure 3:**
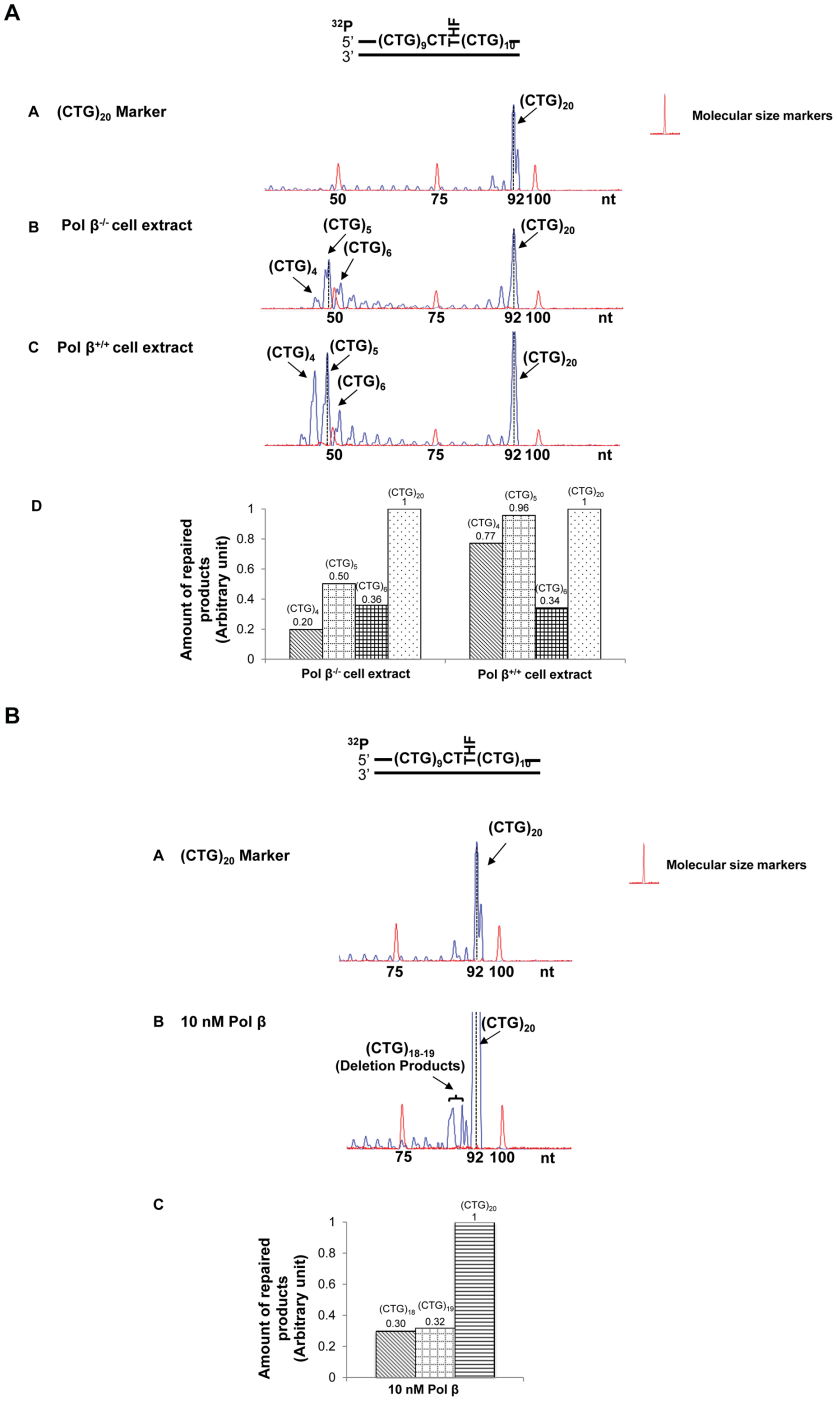
CTG repeat deletions induced by an abasic lesion in the middle of (CTG)_20_ repeats. (**A**) A substrate containing (CTG)_20_ repeats with a THF, inserted for substituting the guanine of the tenth CTG, was incubated with 60 µg cell extracts of pol β^−/−^ or pol β^+/+^ MEFs for 30 min. Panel **A** represents the result of PCR amplification of a DNA marker with (CTG)_20_ repeats. Panels **B** and **C** illustrate the results from BER of the THF residue mediated by pol β^−/−^ and pol β^+/+^ MEFs extracts. Panel **D** represents the quantitative analysis of the results of panels **B** and **C**. (**B**) The repair of a THF in the middle of (CTG)_20_ repeats was performed by BER reconstituted with 10 nM purified pol β (panel **B**). Panel **A** is the result of PCR amplification of a (CTG)_20_ repeat-containing marker, and panel **C** illustrates the quantitative analysis of the results from panel **B**. BER reactions and repeat sizing analysis were performed under the conditions described in the Materials and Methods. Substrates were ^32^P-labeled at the 5′-end of the damaged strand as indicated. Size standards are indicated.

**Figure 4:**
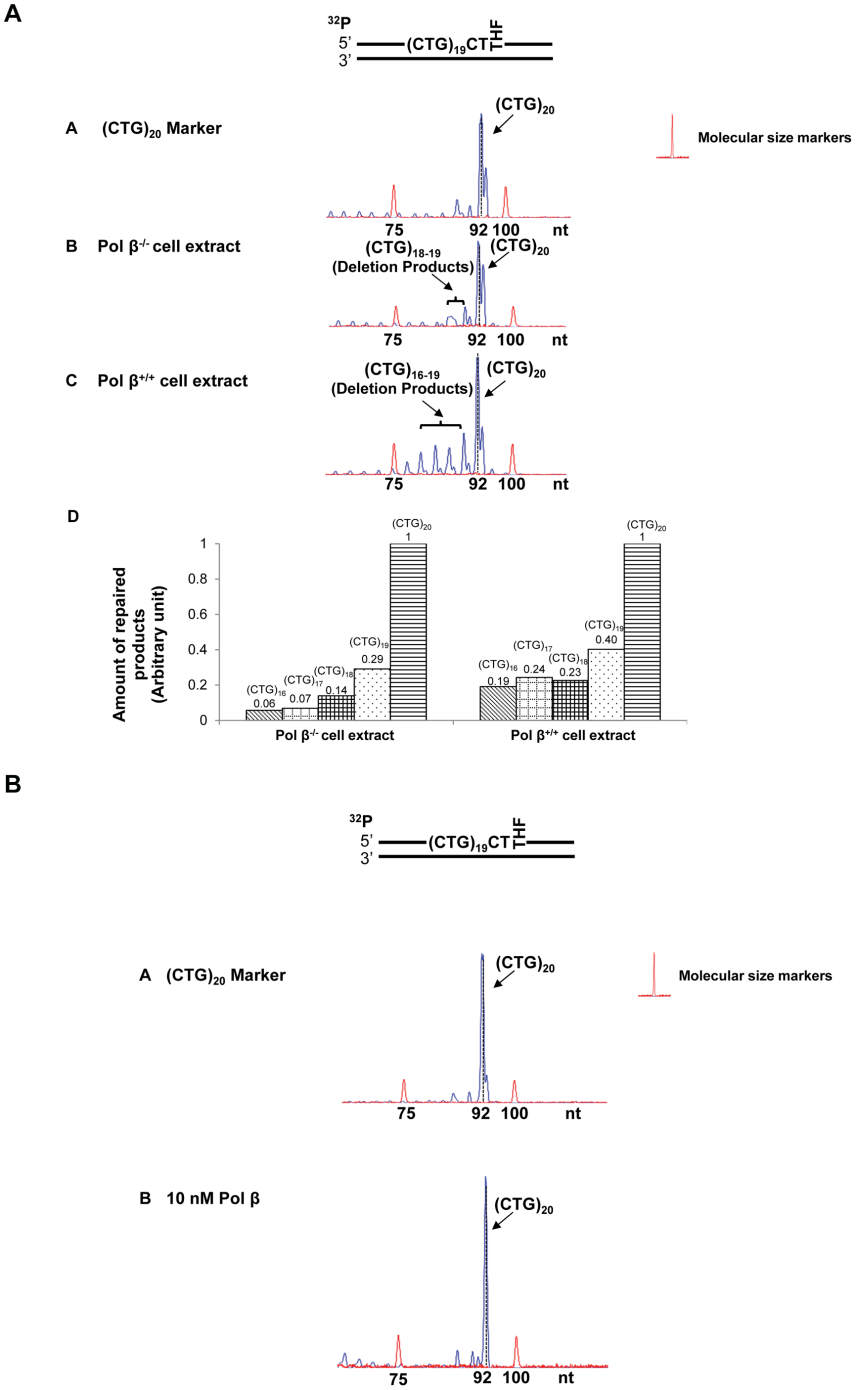
CTG repeat instability resulting from an abasic lesion at the 3′-end of (CTG)_20_ repeats. (**A**) A substrate containing (CTG)_20_ repeats with a THF that substituted the guanine of the twentieth CTG was incubated with 60 µg cell extracts of pol β^−/−^ or pol β^+/+^ MEFs (panels **B** and **C** ) for 30 min. Panel **A** represents the result of PCR amplification of a DNA marker with (CTG)_20_ repeats. Panel **D** represents the quantitative analysis of the results of panels **B** and **C**. (**B**) The repair of a THF at the 3′-end of (CTG)_20_ repeats was performed by BER reconstituted with 10 nM purified pol β (panel **B**). Panel **A** is the result of PCR amplification of a (CTG)_20_ repeat-containing marker without any DNA damage. Substrates were ^32^P-labeled at the 5′-end of the damaged strand as indicated. Size standards are indicated.

**Figure 5:**
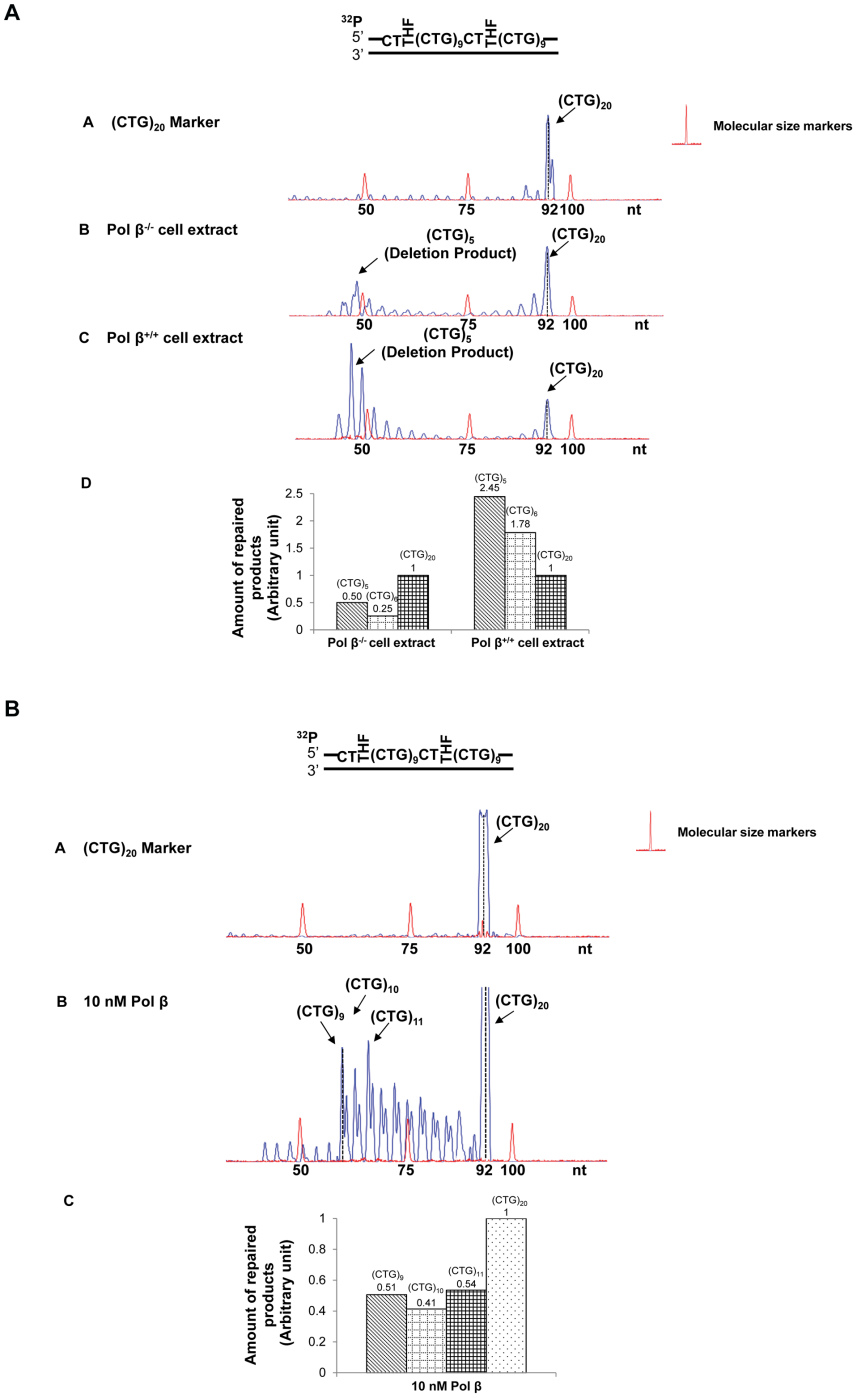
CTG repeat deletions from the abasic lesions located at the 5′-end and in the middle of (CTG)_20_ repeats. (**A**) A substrate containing (CTG)_20_ repeats with two THF residues that substituted the guanines of the first and the tenth CTG, was incubated with 60 µg cell extracts of pol β^−/−^or pol β^+/+^ MEFs for 30 min (panels **B** and **C**). Panel **A** represents the result of PCR amplification of a DNA marker with (CTG)_20_ repeats. Panel **D** represents the quantitative analysis of the results of panels **B** and **C**. (**B**) The repair of two THF residues was performed by BER reconstituted with 10 nM purified pol β (panel **B**). Panel **A** illustrates the result of PCR amplification of a (CTG)_20_ repeat-containing marker without any DNA damage. BER reactions were performed under the conditions described in the Materials and Methods. Substrates were ^32^P-labeled at the 5′-end of the damaged strand as indicated. Size standards are indicated.

The effects of an abasic lesion at these locations on the instability of (CTG)_20_ repeats during BER were initially determined with cell extracts made from pol β^−/−^ or pol β^+/+^ MEFs (Figure 2A, 3A, 4A, 5A), and were verified by reconstituted BER (Figure 2B, 3B, 4B, 5B). The results revealed that a lesion located at the 5′-end of (CTG)_20_ repeats resulted in a (CTG)_21_ expansion product through BER mediated by pol β^−/−^ and pol β^+/+^cell extracts (Figure 2A, panels B and C). The expansion product was also generated by BER reconstituted with10 nM purified pol β in the presence of APE1, FEN1, and LIG I (Figure 2B, panel B). Quantitative analysis showed that the ratio between the amount of (CTG)_21_ expansion product and that of (CTG)_20_ unexpanded products was increased from 3.5 to 6 by the presence of pol β during BER (Figure 2A, panel D and Figure 2B, panel C). This indicates that pol β promoted repeat expansion during BER of an abasic lesion at the 5′-end of the damaged strand. PCR amplification of a DNA marker without any damage gave no repeat expansions or deletions (Figure 2A and 2B, panel A). In addition, PCR amplification of a (CTG)_20_-containing substrate with an intact or APE1-preincised abasic site, failed to produce any amplified products (Figure S5). The results demonstrate that the expansion product was from BER rather than from PCR artifacts. In conclusion, our results indicated that a base lesion located at the 5′-end of (CTG)_20_ led to repeat expansion through BER.

Interestingly, we found that cell extract-based BER of an abasic site located in the middle resulted in deletion products with (CTG)_4_-(CTG)_6_ repeats (Figure 3A, panels B and C). Quantitative analysis demonstrated that pol β increased (CTG)_4_ and (CTG)_5_ deletion products by about 2- to 4-fold, but did not affect the production of (CTG)_6_ deletion product (Figure 3A, panel D). Surprisingly, reconstituted BER of a lesion in the middle of the repeat tract only resulted in small deletion products containing (CTG)_18_ and (CTG)_19_ repeats (Figure 3B, panel B). These results suggest that the large size deletions from cell extracts involve other repair enzymes/proteins in addition to the BER core enzymes, APE1, pol β, FEN1, and LIG I.

Cell extract-based BER of a 3′-end abasic lesion resulted in small deletion products containing (CTG)_16_ to (CTG)_19_ repeats (Figure 4A, panels B and C). The amount of deletion products was increased by approximately 3-fold in pol β^+/+^cell extracts (Figure 4A, panel D). However, reconstituted BER of the damaged products gave neither deletion nor expansion (Figure 4B). This indicated that the repair of a 3′-end base lesion by the core BER enzymes was not sufficient to cause CTG repeat deletions. This further suggests that deletions may essentially be mediated by the cooperation between core BER enzymes and other repair proteins that can shorten CTG repeats from the 3′-end of the damaged strand.

For the scenario in which two base lesions located at both the 5′-end and in the middle of the repeat, large deletion products containing (CTG)_5_ to (CTG)_10_ repeats were detected during cell extract-based and reconstituted BER (Figure 5A, panels B and C, and Figure 5B). Quantitative analysis indicated that deletions were increased by 5- to 7-fold in the presence of endogenous pol β (Figure 5A, panel D). All these results indicate an active role of pol β in promoting both expansions and deletions (Figure 2A, 3A, 4A, 5A). Interestingly, for all the positions, base lesions induced CTG repeat deletions and expansions in pol β^−/−^ cell extracts. Absence of pol β also facilitated the formation of (CTG)_18_ and (CTG)_19_ repeat deletion products (Figure 5A) suggesting a role of pol β-independent BER pathways in modulating both small and large size of TNR deletions and expansions.

It should be noted that the size of both the (CTG)_20_ size marker and the (CTG)_20_ unexpanded repaired products was calculated by DNA fragment analysis to be 92 nt which was 8 nt shorter than its actual length of 100 nt. This is because the (CTG)_20_-containing size marker and repaired products contain stretches of CTG repeats, and the standards for calculating the sizes of DNA fragments contain random sequences. Such difference in DNA sequences resulted in a difference between the mobility of the CTG repeat-containing DNA fragments and that of random sequence DNA fragments during capillary electrophoresis. This led to the difference between the calculated size of a DNA fragment and its actual size.

### Various sizes of Hairpins Formed on the Damaged and Template Strands of (CTG)_20_ Repeats

Because the formation of hairpin structures has been proposed as the basis underlying TNR instability [13], [27], the propensity of a base lesion at specific positions to lead to CTG repeat deletion or expansion could be due to the formation of hairpins at different locations in the repeat tract that favors deletion or expansion. To test this idea, we examined the formation of hairpins on both strands of the (CTG)_20_ repeat-containing substrates after APE1 5′-incision of a THF residue located at different positions in the CTG repeat tract, using Mung Bean Nuclease, the enzyme that preferentially cleaves at a single-stranded hairpin loop as well as at the sites with mismatched base-pairs in the stem region of a hairpin.

**Figure 6:**
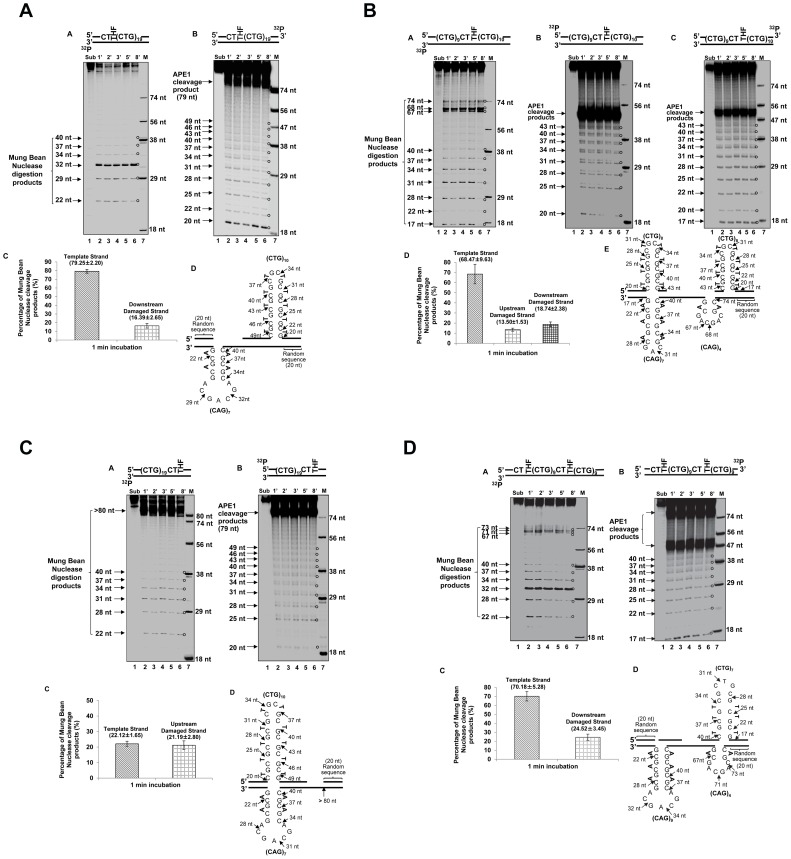
The formation of hairpins resulting from an abasic lesion located at different positions of (CTG)_20_ repeats. The hairpins formed on both the damaged and template strands of the (CTG)_20_ repeat-containing substrate with a base lesion located at different positions of repeat tracts, were probed by Mung Bean Nuclease digestion under the conditions described in the Materials and Methods. The results of hairpin probing from a damage located at the 5′-end, in the middle, or at the 3′-end of the repeat tract are illustrated in (**A**), (**B**), and (**C**), respectively. The results from the damages located at both the 5′-end and in the middle of the repeats are illustrated in (**D**). The relative amount of hairpins was illustrated as the percentage of Mung Bean Nuclease cleavage products. The percentage of Mung Bean Nuclease cleavage products for the damaged strand were calculated by the amount of Mung Bean Nuclease cleavage products that accounted for the formation of hairpin over the total amount of APE1 cleavage products at 1-min interval of enzymatic digestion of hairpins. The percentage of Mung Bean Nuclease cleavage products that represent the formation of a template hairpin induced by the damage at the 5′-end, in the middle, at the 3′-end, and at both the 5′-end and the middle of (CTG)_20_ repeat, was calculated based on 55%, 50%, 56% and 75% of the total amount of the template strand, respectively. A deduced hairpin is illustrated schematically along with specific nuclease digestion sites as indicated. Lane **1** represents the undigested substrates. Lanes **2−6** represent the digestion products generated at various time intervals. Lane **7** represents a series of synthesized size markers (M) for illustrating the size of the digestion products.

For the substrate containing a THF at the 5′-end, the cleavage by Mung Bean Nuclease on the template strand resulted in products of 22 nt, 29 nt, 32 nt, 34 nt, 37 nt, and 40 nt (Figure 6A, panel A). The cleavage pattern indicated the formation of a (CAG)_7_ hairpin with a loop constituted by (CAG)_3_ repeats and a stem consisting of two pairs of CAG repeats (Figure 6A, panel D). The hairpin was located adjacent to the 3′-end flanking region of the template strand. The nuclease cleavage on the damaged strand resulted in products with 20 nt, 22 nt, 25 nt, 28 nt, 31 nt, 34 nt, 37 nt, 40 nt, 43 nt, 46 nt, and 49 nt (Figure 6A, panel B), indicating the formation of a (CTG)_10_ repeat-containing hairpin with a loop composed of two CTG repeats and a stem containing four pairs of CTG repeats (Figure 6A, panel D). The hairpin was adjacent to the 3′-side random sequence of the damaged strand.

For a lesion located in the middle of (CTG)_20_ repeats, the nuclease cleavage on the template strand led to products with 22 nt, 28 nt, 31 nt, 34 nt, 37 nt, 40 nt, 67nt, 68 nt, and 74 nt (Figure 6B, panel A). This cleavage pattern indicates the coexistence of two hairpins on the template strand with seven repeats in between them. One was a (CAG)_7_-repeat containing hairpin adjacent to the 3′-end flanking region and composed of a loop with one CAG and a stem consisting of three pairs of CAG repeats. The other was a (CAG)_4_-repeat-containing hairpin that was two CAG repeats away from the 5′-end flanking region, and contained a loop with two repeats and a short stem with only one pair of CAG repeats (Figure 6B, panel E). For the damaged strand, hairpins forming on both the upstream strand and the downstream strand were probed. The nuclease cleavage on the upstream strand resulted in products with 20 nt, 25 nt, 28 nt, 31 nt, 34 nt, 37 nt, 40 nt, and 43 nt (Figure 6B, panel B), indicating the formation of a (CTG)_8_ repeat hairpin with a loop containing two CTG repeats and a stem with three pairs of repeats (Figure 6B, panel E). The nuclease cleavage on the downstream CTG repeats resulted in products with 20 nt, 22 nt, 25 nt, 28 nt, 31 nt, 34 nt, 37 nt, 40 nt, and 43 nt (Figure 6B, panel C), indicating the formation of a downstream (CTG)_8_ hairpin adjacent to the 3′-flanking region (Figure 6B, panel E).

For a THF located at the 3′-end of (CTG)_20_ repeats, Mung Bean Nuclease cleavage on the template strand resulted in products with 22 nt, 28 nt, 31 nt, 34 nt, 37 nt, and 40 nt (Figure 6C, panel A), indicating the formation of a (CAG)_7_ repeat hairpin on the template strand adjacent to the 3′-flanking region. The nuclease cleavage also resulted in products with larger size (>80 nt). This indicated that the nuclease cleavage in the random sequence flanking region of the template. This may be because of transient dissociation of the 20 nt random DNA sequence from its template after APE1 5′-incision of the THF residue on the damaged strand. This resulted in a single strand region in the template strand that was subsequently captured and cleaved by the nuclease. The nuclease digestion on the damaged strand resulted in products with 20 nt, 25 nt, 31 nt, 34 nt, 37 nt, 40 nt, 43 nt, 46 nt, and 49 nt (Figure 6C, panel B), indicating the existence of a (CTG)_10_ hairpin adjacent to the 5′-side random sequence region with a loop containing two CTG repeats and a stem containing (CTG)_8_ (Figure 6C, panel D).

Finally, for the substrate that contained two base lesions at both the 5′-end and in the middle of the damaged strand, the nuclease cleavage on the template strand resulted in two groups of products. One group contained products of 22 nt, 28 nt, 32 nt, 34 nt, 37 nt, and 40 nt, and the other contained products of 67 nt, 71 nt, and 73 nt (Figure 6D, panel A). This indicates the formation of both (CAG)_9_ and a (CAG)_4_ repeat-containing hairpin on the template strand. As illustrated in panel D of Figure 6D, the (CAG)_9_ hairpin consisted of a loop with (CAG)_3_ repeats and a stem with three pairs of mismatched CAG repeats, and the (CAG)_4_ hairpin contained a loop with two CAG repeats and a stem with one pair of mismatched CAG repeats. The enzymatic cleavage on the damaged strand of the substrate generated products with 22 nt, 25 nt, 28 nt, 31 nt, 34 nt, 37 nt, and 40 nt (Figure 6D, panel B), indicating the formation of a (CTG)_7_ hairpin with a loop containing only one CTG and a stem with three pairs of CTG repeats (Figure 6D, panel D).

Quantitative analysis of Mung Bean Nuclease cleavage products from all of the substrates showed that approximately 16.39±2.65% to 79.25±2.20% of cleavage products were generated from the template strand, the upstream and downstream strands (panel C of Figure 6A, 6C, and 6D; panel D of Figure 6B), indicating that the formation of hairpins during BER is significant.

It also should be noted that a 17 nt product cleavage product was observed during probing of the hairpins induced by a base lesion in the middle or at both the 5′-end and in the middle of (CTG)_20_ repeats (Figure 6B, panels A and C; Figure 6D, panel B). This indicated that Mung Bean Nuclease also made the cleavage in the random sequence regions that flanked the repeats. This could be due to transient dissociation of the random sequence strand from its template strand after Mung Bean Nuclease made the cleavage at the base of hairpins. The dissociated random sequence strand was then captured and cleaved by the enzyme.

To verify the specificity of Mung Bean Nuclease, we examined the enzyme cleavage on a substrate containing a template hairpin composed of a loop of six adenosines and a stem with 15 nt-matched base pairs. The results showed that the enzyme only specifically cleaved at the ssDNA loop region of the hairpin (Supplemental Figure S2). We failed to detect any cleavage products on both the damaged strand and the template strand of a random sequence substrate (Supplemental Figure S3). We also failed to detect Mung Bean Nuclease cleavage products on all of the (CTG)_20_ repeat substrates in the absence of APE1 (Supplemental Figure S4). These results indicated that the formation of hairpins is CTG repeat-specific and exclusively dependent on ssDNA breaks.

### Pol β Multi-nucleotide Gap-filling DNA Synthesis during BER of a THF Residue Located at Different Sites of (CTG)_20_ Repeats

Because our previous study suggested that disruption of pol β and FEN1 coordination during long-patch BER led to TNR expansion [27], and our current results also indicated that pol β promoted TNR deletions (Figures 3, 4, 5), we asked whether the positioning effects of a base lesion on CTG repeat instability could be due to different efficiencies of pol β DNA synthesis and FEN1 flap cleavage activity in the context of various numbers, sizes, and positions of hairpin structures.

**Figure 7:**
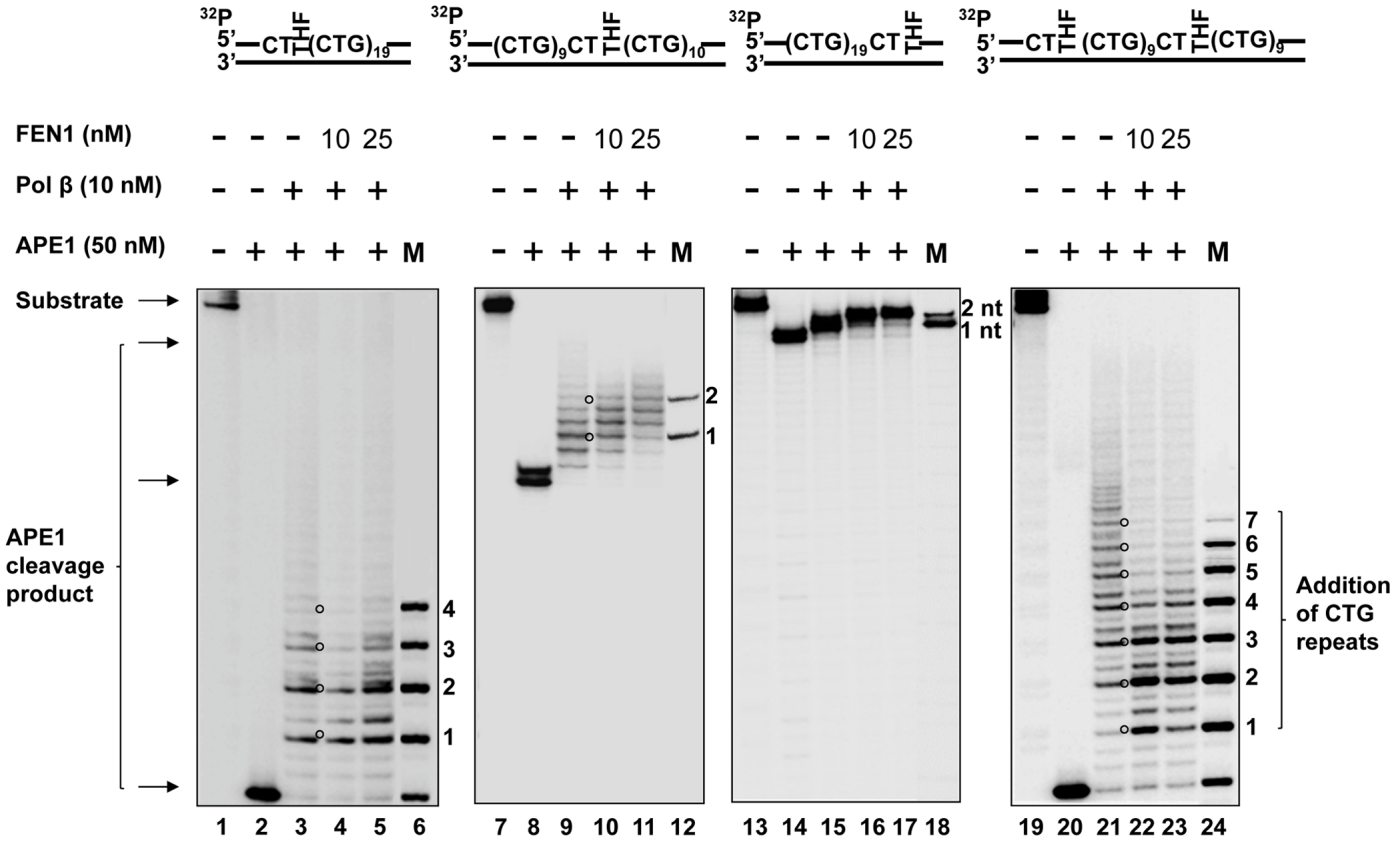
Pol β DNA synthesis during BER of an abasic lesion located at different sites of (CTG)_20_ repeats. Pol β DNA synthesis with the substrates containing one or two THF residues located at the 5′-end, or/and in the middle, or at the 3′-end of (CTG)_20_ repeats was determined in the presence of 10 nM of pol β along with 10 nM and 25 nM FEN1. Lanes **1, 7, 13,** and **19** correspond to substrates only. Lanes **2, 8, 14,** and **20** correspond to reaction mixtures with 50 nM APE1. Lanes **3−5**, **9−11**, **15−17,** and **21−23** correspond to reaction mixtures with 10 nM pol β in the absence or the presence of 10 nM and 25 nM FEN1. Lanes **6**, **12**, **18**, and **24** correspond to a series of synthesized size markers (M) for illustrating the size of pol β DNA synthesis products. Substrates were ^32^P-labeled at the 5′-end of their damaged strands. Substrates are illustrated schematically above the gel.

To address this question, we initially characterized pol β DNA synthesis during the repair of a THF located at the 5′-end, in the middle, and at the 3′-end of (CTG)_20_ repeats as well as during the repair of the two damages located at the 5′-end and in the middle. The results revealed that in the absence or the presence of 10 nM and 25 nM FEN1, 10 nM pol β mainly inserted three CTG repeats for repairing the 5′-end THF group (Figure 7, lanes 3, 4, 5) and four repeats for repairing the base lesions located at both the 5′-end and in the middle of the repeats (Figure 7, lanes 21, 22, 23). However, the same concentration of the enzyme only caused insertion of two repeats for repairing the damage in the middle of the repeat tract (Figure 7, lanes 9, 10, 11), and one or two nucleotides in repairing the 3′-end base damage (Figure 7, lanes 15, 16, 17). The results indicated that pol β mainly performed limited multi-nucleotide DNA synthesis for repairing a base lesion in the middle or at the 3′-end of CTG repeat tracts, but exhibited efficient DNA synthesis for repairing base lesions located at the 5′-end or at both the 5′-end and in the middle of the repeats. It is possible that efficient DNA synthesis of pol β leads to expansions, whereas inefficient DNA synthesis causes deletions through coordination with FEN1 cleavage of CTG flaps. Interestingly, we observed that FEN1 cleavage slightly reduced pol β DNA synthesis by 1 to 3 repeat units during BER of an abasic site located at both the 5′-end and in the middle of (CTG)_20_ repeats (Figure 7, lanes 22–23). This may be due to FEN1 binding to a repair intermediate with a 5′-CTG flap attached to a downstream hairpin that dislodged pol β from the intermediate, thereby inhibiting DNA synthesis by the polymerase. This was also demonstrated by our recent finding showing that FEN1 inhibited pol β DNA synthesis in the presence of a (CTG)_7_ flap, but failed to affect pol β activity in the absence of the flap [36].

### FEN1 Cleavage on CTG Repeat Flaps during the Repair of a Base Lesion at Various Locations of (CTG)_20_ Repeats

Because FEN1 alternate cleavage for processing the 5′-end of a hairpin is critical for TNR expansion [27], and repeat deletions during BER requires a cleavage of repeats for shortening repeat length, we reasoned that FEN1 flap cleavage activity plays a critical role in mediating both repeat expansions and deletions during the repair of an abasic lesion located at specific sites in (CTG)_20_ repeats.

**Figure 8:**
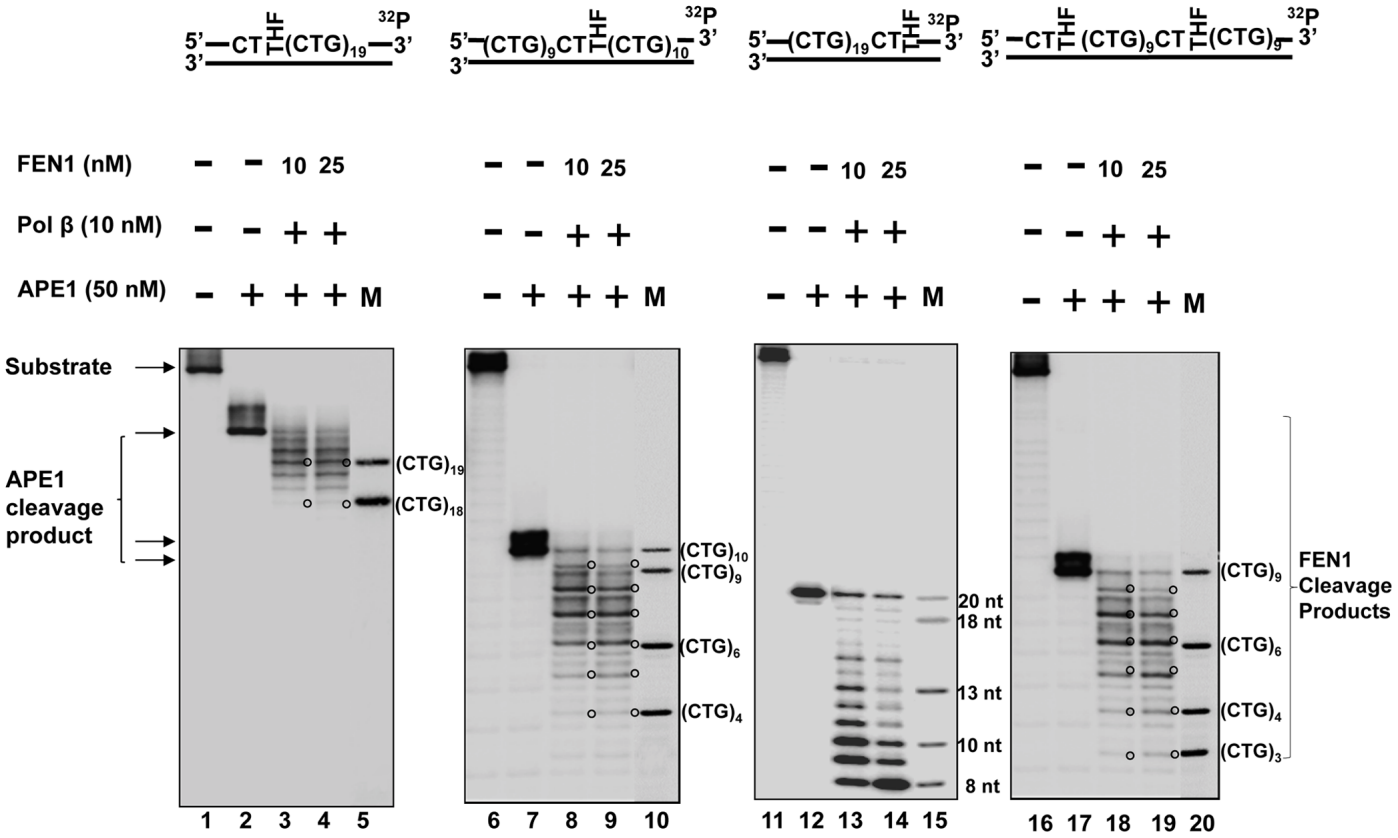
FEN1 cleavage during BER of an abasic lesion located at various sites of (CTG)_20_ repeats. Substrates containing one or two THF residues located at the 5′-end, or/and in the middle, or at the 3′-end of (CTG)_20_ repeats were pre-incubated with 50 nM APE1. FEN1 cleavage activity was determined in the presence of 10 nM and 25 nM FEN1 plus 10 nM pol β under the conditions described in the Materials and Methods. Lanes **1**, **6**, **11,** and **16** correspond to substrates only. Lanes **2**, **7**, **12,** and **17** correspond to reaction mixtures with 50 nM APE1. Lanes **3−4**, **8−9**, **13−14,** and **18−19** correspond to reaction mixtures with 10 nM and 25 nM FEN1 with 10 nM pol β. Lanes **5**, **10**, **15**, and **20** correspond to a series of synthesized size markers (M) for illustrating the size of FEN1 cleavage products. Substrates were ^32^P-labeled at the 3′-end of their damaged strands. Substrates are illustrated schematically above the gel.

To test this idea, we determined FEN1 flap cleavage on the substrates with a THF at different locations of (CTG)_20_ repeats in the presence of 10 nM pol β (Figure 8). The results revealed that FEN1 removed two CTG repeats for repairing a 5′-end THF and six repeats for repairing a THF in the middle and for repairing two THF residues (Figure 8, lanes 3−4, lanes 8−9, and lanes 18−19). However, FEN1 cleaved up to 12 nucleotides in the 3′-side random sequence region in a stepwise manner for repairing a THF located at the 3′-end of the repeat tract (Figure 8, lanes 13−14). This indicated that FEN1 cleaved relatively larger sizes of repeats for repairing the base lesion located in the middle of the repeat tracts than for repairing the damage at the 5′-end.

## Discussion

In this study, we provide the first evidence that CTG repeat expansions and deletions can be induced by oxidative DNA damage (Figure 1) and mediated through BER (Figure 2–5). We demonstrate that the repeat instability is correlated with the positions and the numbers of DNA base lesions in repeat tracts. We further demonstrate that the positioning effect is mediated by the formation of multiple hairpins with varying sizes on both the template and damaged strands of (CTG)_20_ repeats (Figure 6). This is accomplished by different efficiencies of pol β DNA synthesis and FEN1 flap cleavage (Figure 7−8). Pol β exhibited more efficient DNA synthesis (Figure 7, lanes 3−5) than FEN1 flap cleavage (Figure 8, lanes 3−4) to repair damage located at the 5′-end of the repeats, thereby leading to expansion. In contrast, FEN1 exhibited more efficient cleavage (Figure 8, lanes 8−9) than pol β DNA synthesis (Figure 7, lanes 9, 10, 11) to repair a damage located in the middle of CTG repeats, leading to one or two repeat deletions. For the two damages located at both the 5′-end and in the middle of CTG repeats, high efficiency of pol β DNA synthesis displaces the repeat strand in between the two damaged sites from the template, and high efficiency of FEN1 cleavage removes more repeats. The synergistic effect of efficient pol β and FEN1 activity leads to large deletions. For a base lesion located at the 3′-end of the repeats, the repair event is carried out in the context of a random DNA sequence. Pol β and FEN1 activity fail to affect the repeat instability.

**Figure 9:**
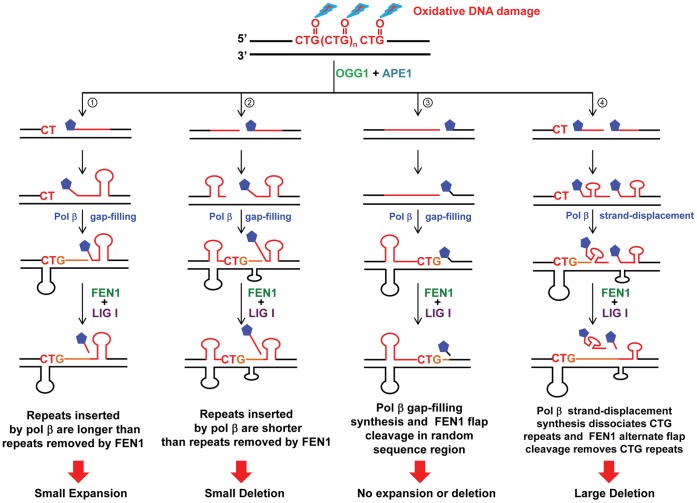
Hypothetical models illustrating CTG repeat instability governed by the positions of oxidative DNA base lesions through pol β and FEN1. Oxidative stress can result in an oxidized DNA base lesion, 8-oxoG that is located either at the 5′-end, in the middle, or at the 3′-end of CTG repeats. Removal of the damaged site by OGG1 leaves an abasic site that is subsequently 5′-incised by APE1 generating ssDNA breakage, results in the formation of multiple hairpins on repeat tracts. A base lesion that occurs at the 5′-end of the repeats allows the formation of a multi-nucleotide gap and subsequently a 5′-hairpin along with a template hairpin. Pol β passes through the template hairpin and synthesizes more repeats than those removed by FEN1, thereby causing repeat expansions (sub-pathway **1**). A base lesion located in the middle of CTG repeats results in the formation of an upstream hairpin and a downstream hairpin in the damaged strand along with two template hairpins. The upstream hairpin leads to inefficient pol β DNA synthesis that inserts one or two repeats, but allows FEN1 to remove more repeats than those synthesized by pol β, leading to small repeat deletions (sub-pathway **2**). A base damage located at the 3′-end of repeats immediately adjacent to the random sequence region, allows the formation of a large upstream hairpin and a template hairpin. Pol β inserts one to three nucleotides to fill a single-nucleotide gap. In this scenario, repeats in the upstream strand cannot be processed by repair activities, thereby leading to maintenance of repeat length (sub-pathway **3**). When two base lesions simultaneously occur at the 5′-end and in the middle of CTG repeat tracts, two hairpins in the damaged strand and two template hairpins would form. This leads to efficient pol β DNA synthesis that can strand-displace the hairpin in between two damage sites, resulting in loss of large numbers of repeats. Efficient FEN1 cleavage of the 5′-downstream CTG repeats leads to additional shortening of repeats. The cooperation between pol β strand-displacement synthesis and FEN1 cleavage leads to large repeat deletions (sub-pathway **4**).

Our results support the idea that differences in the efficiency of pol β DNA synthesis and FEN1 cleavage underlies the positioning effect of a base lesion on TNR instability. Based on the results, we can suggest hypothetical models that illustrate the positioning effect of a DNA base lesion mediated by pol β and FEN1 during long-patch BER (Figure 9). The paths for inducing TNR instability by a base lesion or a ssDNA break located at the 5′-end, in the middle, and at the 3′-end of CTG repeats are illustrated in sub-pathways 1, 2, and 3 (Figure 9). The path for TNR instability induced by the lesions located at both the 5′-end and in the middle of the repeats is illustrated in sub-pathway 4 (Figure 9).

Substantial expansions of CTG repeats in the 3′-untranslated region of the myotonic dystrophy protein kinase gene have been implicated as the cause of DM1 [37]. Interestingly, CTG repeat deletions have also been identified during replication and transcription in bacterial and human cells [5], [7], [38], and this is proposed as one avenue for DM1 treatment [39]. Therefore, understanding of the mechanisms underlying CTG repeat deletions will help identify new targets for disease treatment. Herein, we observed predominantly large deletions, but also small expansions in the tract of (CTG)_35_ and (CTG)_20_ repeats induced by environmental and endogenous oxidative DNA damaging agents in human cells (Figure 1). This is consistent with the finding that H_2_O_2_ increased the rate of CTG/CAG repeat deletions in bacteria [5]. Our attempt to explore the molecular basis underlying CTG instability revealed that the positions and the numbers of DNA base lesions and/or ssDNA breaks governed the formation of hairpins with varying sizes at specific locations of TNRs. This subsequently modulated the efficiency of BER enzymes, causing either repeat expansions or deletions. Our results suggest that CTG repeat expansions or deletions can be regulated by the interactions between dynamic DNA structures in CTG repeats and BER proteins. We demonstrated that BER mediated by pol β^+/+^ cell extracts led to significant amounts of expansion and deletion products (Figures 2A, 3A, and 4A) when compared to pol β^−/−^ cell extracts, indicating that pol β can promote CTG repeat-expansion and deletion. This further suggests an involvement of pol β-mediated BER in modulating TNR instability.

Interestingly, we observed some expansion and deletion products resulting from the repair of an abasic lesion by pol β^−/−^ cell extracts with the same sizes as the ones from pol β^+/+^ cell extracts (panel B of Figures 2A, 3A, and 4A). This suggests that pol β-independent BER pathways also play an important role in modulating CTG repeat instability. Because pol β-independent BER usually involves replication and other repair DNA polymerases such as pol δ, pol ε [40], and pol λ [41], it appears that these DNA polymerases may substitute pol β to perform effective DNA synthesis to repair base lesions located at various positions in TNRs. Therefore, it will be important to explore the roles of replicative DNA polymerases, pol λ, and other X family polymerases in modulating TNR expansions and deletions during BER of oxidative DNA damage.

Larger sizes of repeats (15 repeats) were deleted through cell extract-based BER than through BER reconstituted with purified BER enzymes (one to ten repeats, Figures 3 and 5). This indicated that BER mediated by MEF cell extracts caused much more severe TNRs deletions than BER reconstituted by the core BER enzymes, suggesting that other DNA repair proteins in cell extracts may facilitate the large repeat deletions. This could result from the activity of other nucleases that cooperate with FEN1 cleavage activity.

This possibility is supported by a recent study showing that a 5′–3′ exonuclease, exonuclease 1 (EXO1) cooperated with FEN1 to remove flaps of CTG and CGG repeats during DNA replication [42]. This suggests that a synergistic effect from EXO1 and FEN1 may promote large TNR deletions. In addition, TNRs might also be processed by 3′–5′exonucleases, such as Mre 11 [43]. This can lead to shortening of the repeats by cleaving the 3′-end of TNR, thereby contributing to large repeat deletions in coordination with EXO1 and FEN1 flap cleavages. Large deletions could also be promoted by stabilizing a template hairpin via hairpin binding proteins, MSH2/MSH3 [44]. This may stimulate pol β hairpin bypass synthesis, resulting in loss of large numbers of repeat units. The roles of various nucleases and hairpin binding proteins in causing large TNR deletions during BER in a coordinated manner need to be elucidated in further studies.

In this study, we also provide the first evidence that a base lesion located at various positions on (CTG)_20_ repeats can result in the formation of multiple hairpins with varying sizes at specific positions on CTG/CAG repeat tracts (Figure 6). The coexistence of multiple hairpin structures on both the template and the damaged strand allowed the formation of a cluster of hairpins that may resemble the clustered slip-outs as described in a recent study [45]. Such clustered slip-outs have been found to act as roadblocks for their repair by hMutSβ (MSH2/MSH3) complexes, and therefore are proposed to be responsible for CTG/CAG repeat expansion. It is conceivable that clustered hairpin structures generated during BER may also function as blockages to promote inefficient BER that ultimately leads to expansions and deletions. Consistent with this notion, we found inefficient activity of pol β DNA synthesis (Figure 7, lanes 9−11), or limited FEN1 cleavage of 5′-hairpins (Figure 8, lanes 3−4) in the presence of multiple hairpins on both DNA strands. The role of BER in governing TNR instability for coordination with MSH2/MSH3 through repairing clustered hairpins must be further elucidated.

In conclusion, we demonstrated for the first time that oxidative DNA damage can lead to both CTG deletions and expansions in human somatic cells and *in vitro*. We found that BER of a base lesion at a specific position on (CTG)_20_ repeats correlates with either deletion or expansion, i.e., a damage at the 5′-end of repeats preferentially leads to repeat expansion, whereas a damage in the middle results in deletions. A lesion located at the 3′-end mainly leads to maintenance of repeat lengths. The positioning effect results from the formation of multiple hairpin structures with varying sizes at different locations on repeat tracts. Hairpins at specific locations lead to different efficiency of pol β DNA synthesis and FEN1 cleavage activity that governs whether the repeats can be expanded or deleted. We propose that the positioning effect of oxidative DNA base damage on TNR instability is a consequence of the interaction between hairpin structures and BER enzymes and cofactors. Our study defines a mechanism underlying oxidative DNA base lesion-induced TNR instability.

## Supporting Information

Figure S1 **Oxidative DNA damage does not alter the length of random DNA sequences in human cells.** (**A**) Plasmids containing a fragment with random DNA sequence that has the same length as (CTG)_35_/(CAG)_35_ repeat-containing fragment (225 nt) were transfected into HEK293-H cells. Cells were subsequently treated with oxidative DNA-damaging agents as described in the Materials and Methods. Panel **A** represents the results from untreated cells. Panels **B**, **C,** and **D** represent the results from the cells treated with KBrO_3_, K_2_CrO_4,_ and H_2_O_2_, respectively. (**B**) Plasmids containing a fragment with random DNA sequence that has the same length as (CTG)_20_/(CAG)_20_ repeat-containing fragment (100 nt) were transfected into HEK293-H cells that were treated with oxidative DNA-damaging agents as described in the Materials and Methods. Panel **A** is the result from untreated cells. Panels **B**, **C,** and **D** represent the results from the cells treated with KBrO_3_, K_2_CrO_4,_ and H_2_O_2_, respectively. Size standards are illustrated.
(TIF)

Figure S2 **Mung Bean Nuclease specifically cleaves the loop region of a stable hairpin.** A substrate containing a template hairpin composed of a loop of six adenines and a stem with 15 nt base pairs was radiolabeled at the 5′-end of its template strand. The substrate was incubated with 0.01 U Mung Bean Nuclease at 0.5- and 1-min time intervals (Lanes **2** and **3**). Lane **1** represents substrate only. Lane **4** represents synthesized markers (M) with 30, 45, 48, 51, 66 nucleotides, respectively.
(TIF)

Figure S3 **No hairpin forms in the context of random sequences.** The formation of a hairpin on both the template strand and the damaged strand of random sequence of a substrate with a THF residue at the 5′-end of its damaged strand was probed by Mung Bean Nuclease digestion. The substrate was radiolabeled at either the 5′-end of its template strand (panel **A**) or the 3′-end of its damaged strand (panel **B**). The substrate was precut by 10 nM APE1 and was incubated with 1 U Mung Bean Nuclease at 1-, 2-, 3-, 5-, 8-min time intervals (lanes **2–6** of panel **A** and lanes **8–12** of panel **B)** under the conditions described in the Materials and Methods. Lane **1** represents the undigested substrate.
(TIF)

Figure S4 **No hairpin forms in the absence of ssDNA breakage.** (**A**) Hairpin formation on both the template strand and the damaged strand of a (CTG)_20_-containing substrate with a THF residue at the 5′-end was probed by Mung Bean Nuclease in the absence of APE1 cleavage. The substrate was radiolabeled at the 3′-end of its template strand (left panel) or its damaged strand (right panel). The substrate was incubated with 1 U Mung Bean Nuclease at 1-, 2-, 3-, 5-, 8-min time intervals. Lane **1** and **8** represent substrate without enzyme digestion. Lane **7** represents synthesized markers (M) with 18, 29, 38, 56, 74 nucleotides. Lane **14** represents synthesized markers with 18, 29, 38, 47, 56, 74 nucleotides. (**B**) The formation of hairpins on the template strand and the damaged strand of a (CTG)_20_-containing substrate with a THF residue in the middle of CTG repeats was probed by Mung Bean Nuclease in the absence of APE1 cleavage under the condition described in (**A**). The substrate was radiolabeled at the 3′-end of its template strand (left panel) or the 5′- or the 3′-end of its damaged strand (middle and right panels). Lanes **1**, **8** and **15** represent substrate only. Lanes **7**, **14** and **21** represent the same synthesized markers (M) described in (**A**). (**C**) Hairpin formation on the template strand and the damaged strand of a (CTG)_20_-containing substrate with a THF residue at the 3′-end of the repeat track was probed by Mung Bean Nuclease in the absence of APE1. The substrate was radiolabeled at the 3′-end of its template (left panel) or the 5′-end of its damaged strand (right panel). The substrate was incubated with 1 U Mung Bean Nuclease under the condition described in (**A**). Lane **1** and **8** represent substrate only. Lane **7** and **14** represent the same synthesized markers (M) described in (**A**). (**D**) Hairpins on the template strand and the damaged strand of (CTG)_20_-containing substrate with two THF residues were probed by Mung Bean Nuclease in the absence of APE1 under the condition described in (**A**). The substrate was radiolabeled at the 3′-end of either its template (left panel) or its damaged strand (right panel). Lane **1** and **8** represent substrate only. Lane **7** and 14 represent the same synthesized markers (M) as used in (**A**).
(TIF)

Figure S5 **PCR amplification of a (CTG)_20_ repeat-containing substrate with an AP site at the 5′-end of the damaged strand with or without APE1 5′-incision.** PCR reactions were performed with the substrate containing a THF residue at the 5′-end with or without APE1 5′-incision under the conditions described in the Materials and Methods. Lane 2 represents the result of PCR amplification of the damage-containing substrate without APE1 5′-incision. Lane 3 represents the result of PCR amplification of the substrate preincised by 50 nM APE1. Lane 4 represents the result of PCR amplification of a (CTG)_20_-contaning marker without any damage. Lane 1 and 5 represent DNA size markers ranging from 100 bp to 1000 bp.
(TIF)

Table S1 Oligonucleotides sequences.
(DOCX)
