# Supplementary figures and images for: Instability of CTG Repeats is Governed by the Position of a DNA Base Lesion through Base Excision Repair
Source: PLoS One. 2013 Feb 26;8(2):e56960. doi: 10.1371/journal.pone.0056960 (PMC3582642; doi:10.1371/journal.pone.0056960)

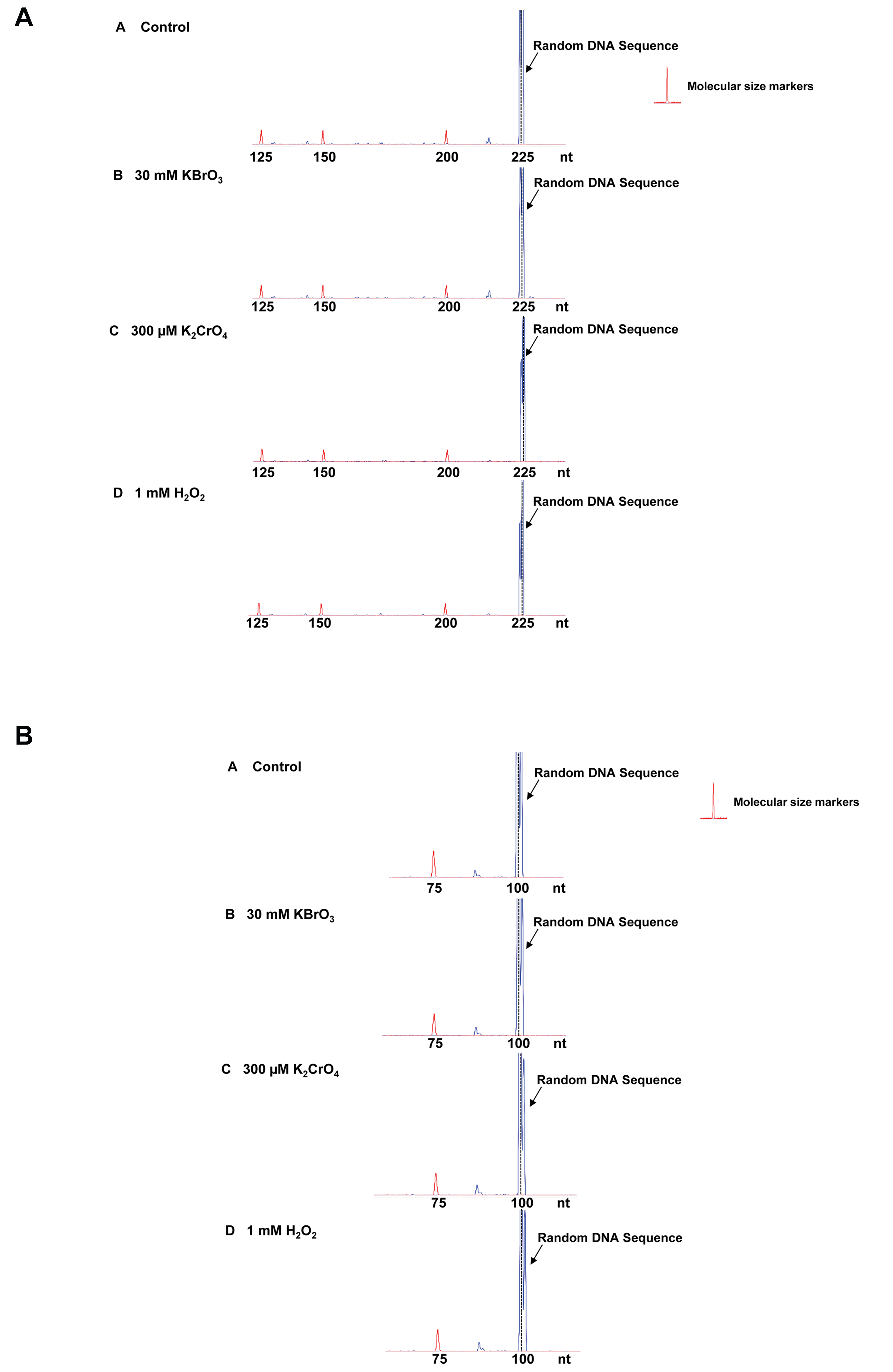

Supplement: Figure S1 — Oxidative DNA damage does not alter the length of random DNA sequences in human cells. (A) Plasmids containing a fragment with random DNA sequence that has the same length as (CTG)35/(CAG)35 repeat-containing fragment (225 nt) were transfected into HEK293-H cells. Cells were subsequently treated with oxidative DNA-damaging agents as described in the Materials and Methods. Panel A represents the results from untreated cells. Panels B, C, and D represent the results from the cells treated with KBrO3, K2CrO4, and H2O2, respectively. (B) Plasmids containing a fragment with random DNA sequence that has the same length as (CTG)20/(CAG)20 repeat-containing fragment (100 nt) were transfected into HEK293-H cells that were treated with oxidative DNA-damaging agents as described in the Materials and Methods. Panel A is the result from untreated cells. Panels B, C, and D represent the results from the cells treated with KBrO3, K2CrO4, and H2O2, respectively. Size standards are illustrated. (TIF) [file pone.0056960.s001.tif]

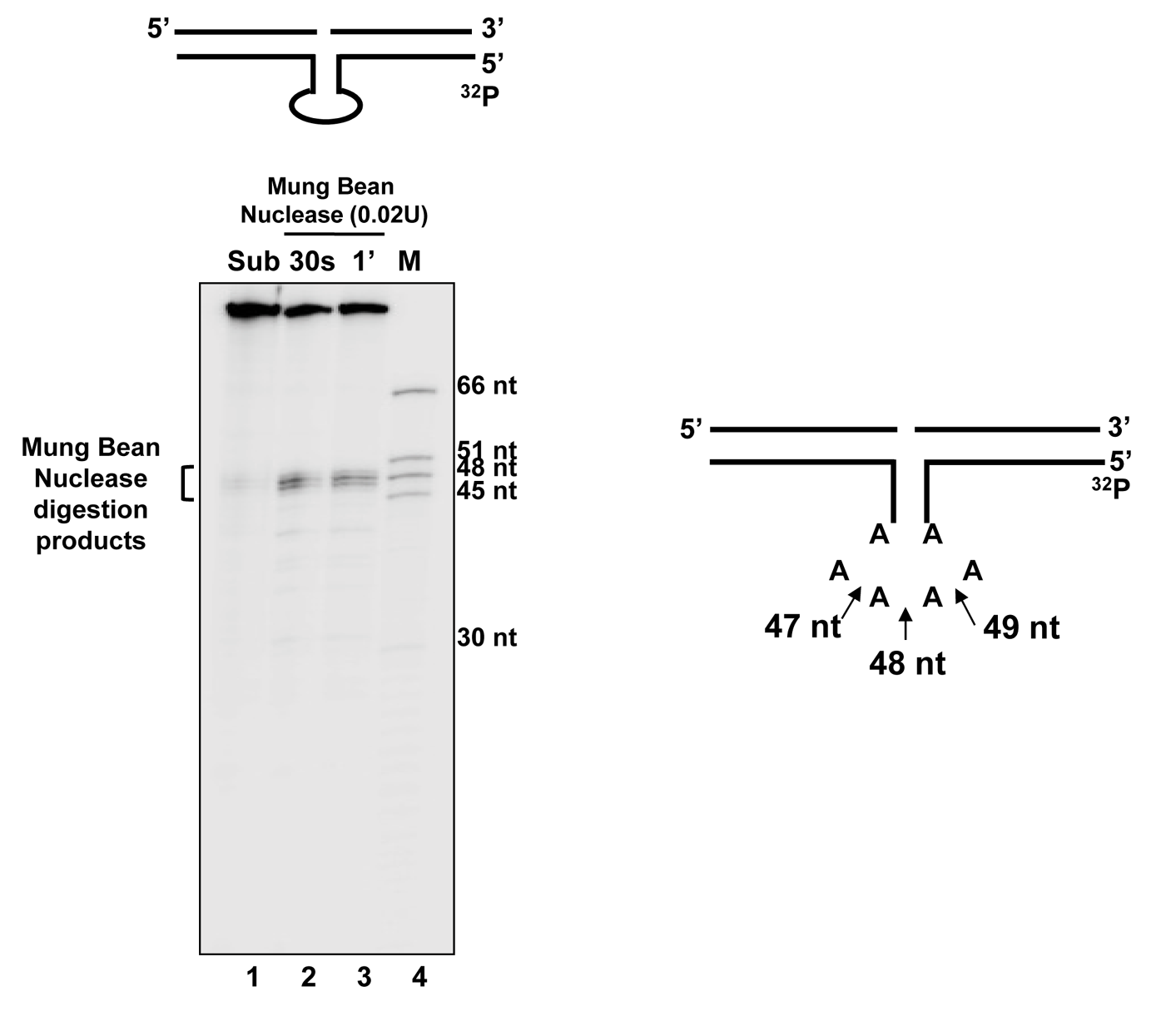

Supplement: Figure S2 — Mung Bean Nuclease specifically cleaves the loop region of a stable hairpin. A substrate containing a template hairpin composed of a loop of six adenines and a stem with 15 nt base pairs was radiolabeled at the 5′-end of its template strand. The substrate was incubated with 0.01 U Mung Bean Nuclease at 0.5- and 1-min time intervals (Lanes 2 and 3). Lane 1 represents substrate only. Lane 4 represents synthesized markers (M) with 30, 45, 48, 51, 66 nucleotides, respectively. (TIF) [file pone.0056960.s002.tif]

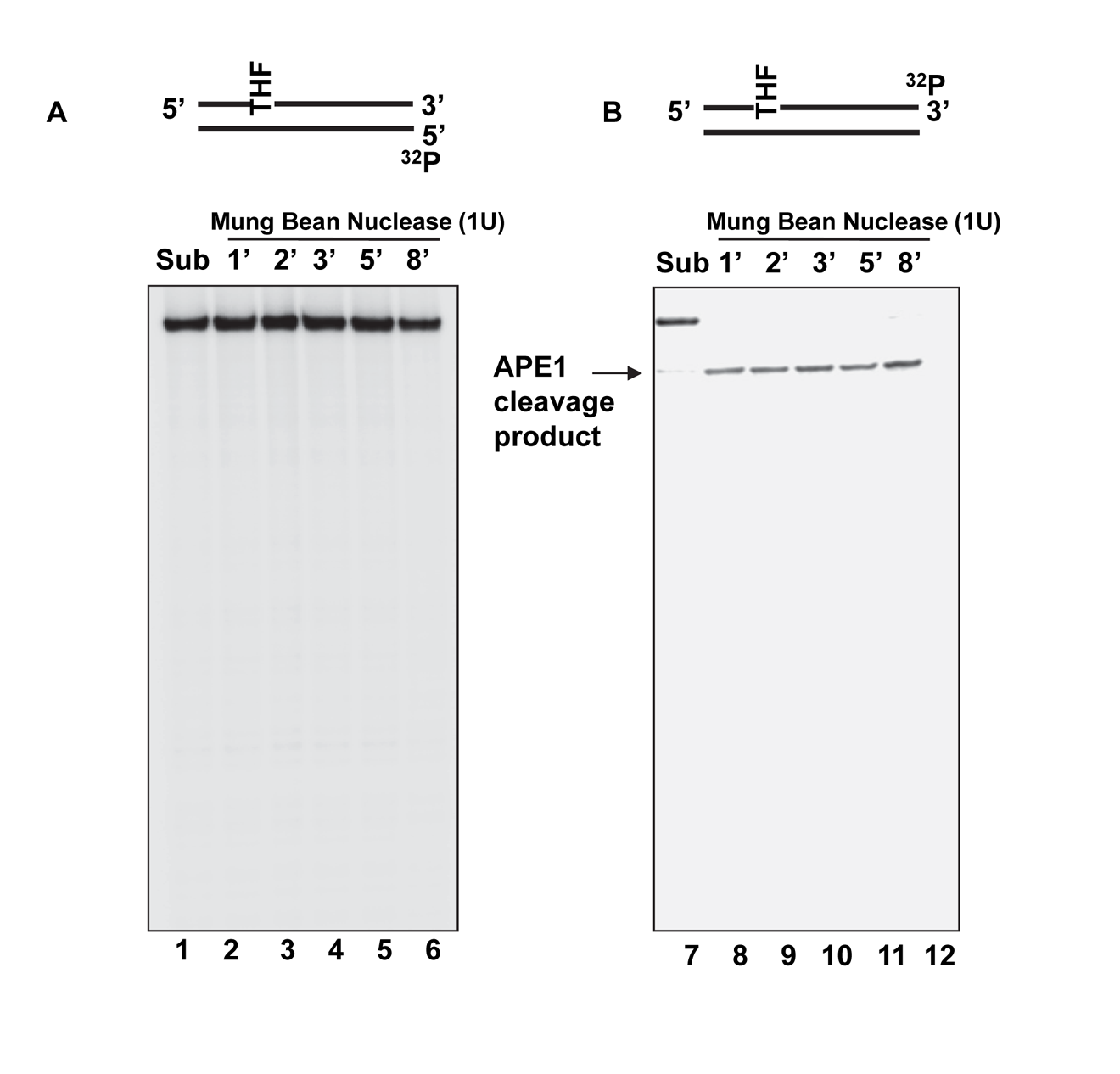

Supplement: Figure S3 — No hairpin forms in the context of random sequences. The formation of a hairpin on both the template strand and the damaged strand of random sequence of a substrate with a THF residue at the 5′-end of its damaged strand was probed by Mung Bean Nuclease digestion. The substrate was radiolabeled at either the 5′-end of its template strand (panel A) or the 3′-end of its damaged strand (panel B). The substrate was precut by 10 nM APE1 and was incubated with 1 U Mung Bean Nuclease at 1-, 2-, 3-, 5-, 8-min time intervals (lanes 2–6 of panel A and lanes 8–12 of panel B) under the conditions described in the Materials and Methods. Lane 1 represents the undigested substrate. (TIF) [file pone.0056960.s003.tif]

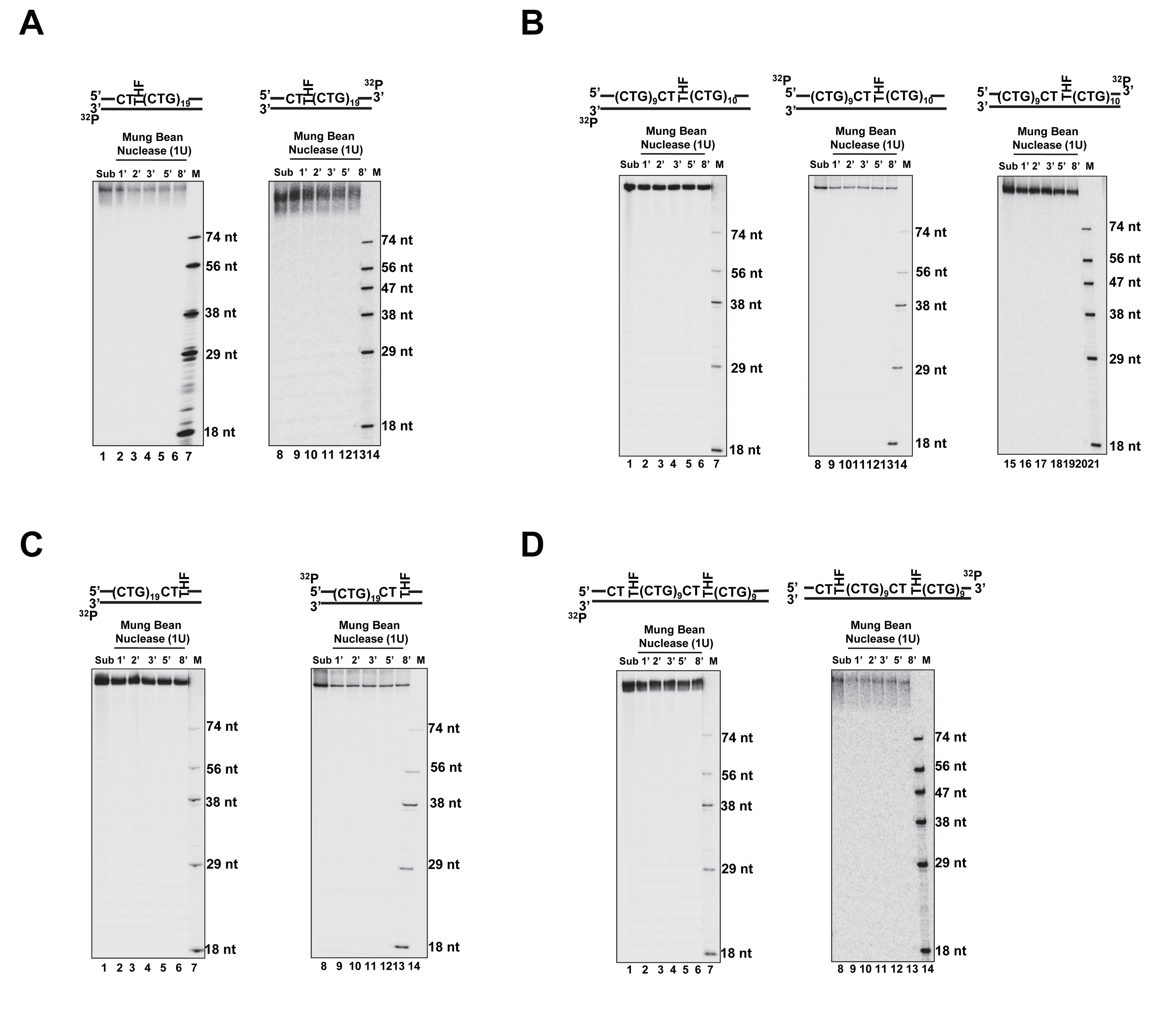

Supplement: Figure S4 — No hairpin forms in the absence of ssDNA breakage. (A) Hairpin formation on both the template strand and the damaged strand of a (CTG)20-containing substrate with a THF residue at the 5′-end was probed by Mung Bean Nuclease in the absence of APE1 cleavage. The substrate was radiolabeled at the 3′-end of its template strand (left panel) or its damaged strand (right panel). The substrate was incubated with 1 U Mung Bean Nuclease at 1-, 2-, 3-, 5-, 8-min time intervals. Lane 1 and 8 represent substrate without enzyme digestion. Lane 7 represents synthesized markers (M) with 18, 29, 38, 56, 74 nucleotides. Lane 14 represents synthesized markers with 18, 29, 38, 47, 56, 74 nucleotides. (B) The formation of hairpins on the template strand and the damaged strand of a (CTG)20-containing substrate with a THF residue in the middle of CTG repeats was probed by Mung Bean Nuclease in the absence of APE1 cleavage under the condition described in (A). The substrate was radiolabeled at the 3′-end of its template strand (left panel) or the 5′- or the 3′-end of its damaged strand (middle and right panels). Lanes 1, 8 and 15 represent substrate only. Lanes 7, 14 and 21 represent the same synthesized markers (M) described in (A). (C) Hairpin formation on the template strand and the damaged strand of a (CTG)20-containing substrate with a THF residue at the 3′-end of the repeat track was probed by Mung Bean Nuclease in the absence of APE1. The substrate was radiolabeled at the 3′-end of its template (left panel) or the 5′-end of its damaged strand (right panel). The substrate was incubated with 1 U Mung Bean Nuclease under the condition described in (A). Lane 1 and 8 represent substrate only. Lane 7 and 14 represent the same synthesized markers (M) described in (A). (D) Hairpins on the template strand and the damaged strand of (CTG)20-containing substrate with two THF residues were probed by Mung Bean Nuclease in the absence of APE1 under the condition described in (A). The substrate was [file pone.0056960.s004.tif]

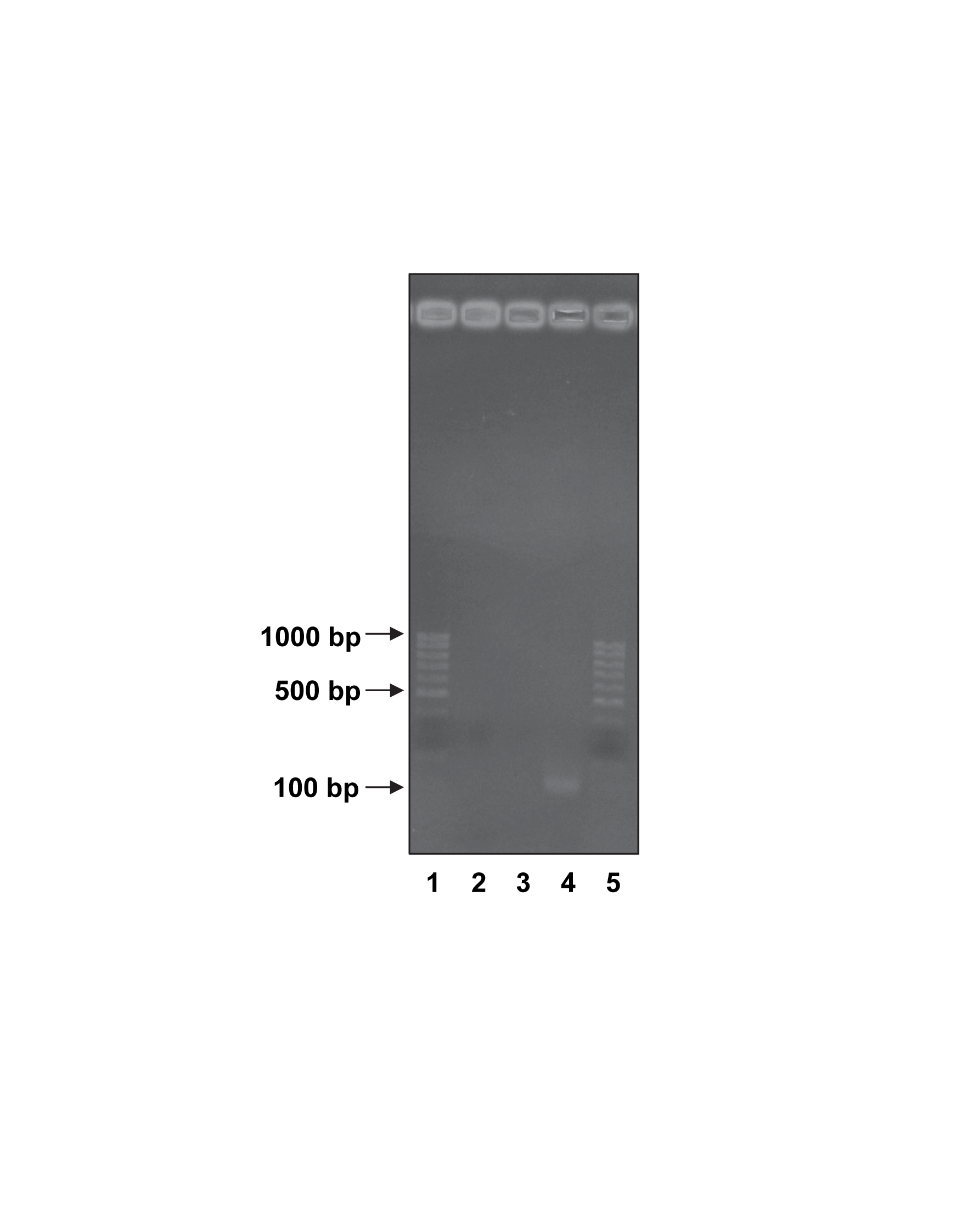

Supplement: Figure S5 — PCR amplification of a (CTG)20 repeat-containing substrate with an AP site at the 5′-end of the damaged strand with or without APE1 5′-incision. PCR reactions were performed with the substrate containing a THF residue at the 5′-end with or without APE1 5′-incision under the conditions described in the Materials and Methods. Lane 2 represents the result of PCR amplification of the damage-containing substrate without APE1 5′-incision. Lane 3 represents the result of PCR amplification of the substrate preincised by 50 nM APE1. Lane 4 represents the result of PCR amplification of a (CTG)20-contaning marker without any damage. Lane 1 and 5 represent DNA size markers ranging from 100 bp to 1000 bp. (TIF) [file pone.0056960.s005.tif]
