# Supplementary material for: Instability of CTG Repeats is Governed by the Position of a DNA Base Lesion through Base Excision Repair
Source: PLoS One. 2013 Feb 26;8(2):e56960. doi: 10.1371/journal.pone.0056960 (PMC3582642; doi:10.1371/journal.pone.0056960)
Supplement: Table S1 — Oligonucleotides sequences. (DOCX) [file pone.0056960.s006.docx]

**Table S1 Oligonucleotides sequences**

| Oligonucleotides | nt | | Sequence (5’-3’) |
| --- | --- | --- | --- |
| Substrates | | | |
| Damaged Strands | | | |
| D1 | **100** | | GCA ATG AGT AAG TCT ACG TA CT **F** CTG CTG CTG CTG CTG CTG CTG CTG CTG CTG CTG CTG CTG CTG CTG CTG CTG CTG CTG TA CGG ATG CTA GAT GAC TCG |
| D2 | **100** | | GCA ATG AGT AAG TCT ACG TA CTG CTG CTG CTG CTG CTG CTG CTG CTG CT **F** CTG CTG CTG CTG CTG CTG CTG CTG CTG CTG TA CGG ATG CTA GAT GAC TCG |
| D3 | **100** | | GCA ATG AGT AAG TCT ACG TA CTG CTG CTG CTG CTG CTG CTG CTG CTG CTG CTG CTG CTG CTG CTG CTG CTG CTG CTG CT **F** TA CGG ATG CTA GAT GAC TCG |
| D4 | **100** | | GCA ATG AGT AAG TCT ACG TA CT **F** CTG CTG CTG CTG CTG CTG CTG CTG CTG CT **F** CTG CTG CTG CTG CTG CTG CTG CTG CTG TA CGG ATG CTA GAT GAC TCG |
| Template | | | |
| T | **100** | CGA GTC ATC TAG CAT CCG TA CAG CAG CAG CAG CAG CAG CAG CAG CAG CAG CAG CAG CAG CAG CAG CAG CAG CAG CAG CAG TA CGT AGA CTT ACT CAT TGC | |
| PCR Primers | | | |
| (CTG)_35_ FP | **23** | 6-FAM CTA GTC CAG TGT GGT GGA ATT GC | |
| (CTG)_35_ RP | **21** | CAT TCT AGA CTC GAG CGG CCG | |
| (CTG)_20_ FP | **22** | 6-FAM-CGA GTC ATC TAG CAT CCG TA CA | |
| (CTG)_20_ RP | **20** | GCA ATG AGT AAG TCT ACG TA | |
| Random sequence FP | **26** | 6-FAM TAA TAC GAC TCA CTA TAG GGA GAC CC | |
| Random sequence RP225 | **27** | AGT GAA AAG TTC TTC TCC TTT GCT AGC | |
| Random sequence RP100 | **25** | GCA TTG TAC TTG TGG ATA GCT TG GC | |
| ^a^ The damaged base is in boldface. F, tetrahydofuran. ^b^ FP, Forward Primer. RP, Reverse Primer. | | | |
